# Diffusion Inverse Filtering: Enhancing Functional Connectivity-Based Pattern Recognition by Counteracting Spatial Smoothing

**DOI:** 10.3390/brainsci16080869

**Published:** 2026-08-16

**Authors:** Yuzeng Xu, Sho Otsuka, Seiji Nakagawa

**Affiliations:** 1 Graduate School of Science and Engineering, Chiba University, Chiba 263-8522, Japan; yuzeng-xu@chiba-u.jp; 2 Center for Frontier Medical Engineering, Chiba University, Chiba 263-8522, Japan; otsuka.s@chiba-u.jp; 3 Graduate School of Engineering, Chiba University, Chiba 263-8522, Japan; 4 Department of Medical Engineering, Faculty of Engineering, Chiba University, Chiba 263-8522, Japan; 5 Med-Tech Link Center, Chiba University Hospital, Chiba 260-8677, Japan

**Keywords:** EEG, brain-computer interface, functional connectivity, volume conduction, spatial smoothing, affective computing, deep learning

## Abstract

**Highlights:**

**What are the main findings?**

- The proposed diffusion inverse filtering (DIF) method performs a spatially informed inverse-diffusion transformation directly on EEG functional-connectivity representations.
- DIF was proposed to perform a spatially informed in-verse-diffusion transformation directly on EEG functional-connectivity representations to counteract spatial smoothing.
- DIF generally achieves higher emotion-classification performance than the original functional-connectivity representations, demonstrating the effectiveness of spatially informed transformation in enhancing the discriminative power of functional-connectivity representations.

**What are the implications of the main findings?**

- DIF provides a practical functional-connectivity-level alternative to conventional signal-level processing and graph-signal processing (GSP) approaches for counteracting spatial smoothing and enhancing EEG functional-connectivity representations.
- Evaluation within an emotion-classification framework and under electrode sparsification suggests that DIF maintains competitive performance with reduced electrode densities and network sizes, supporting its application in connectivity-based brain–computer interface (BCI) systems.

**Abstract:**

**Background/Objectives:** This study proposes Diffusion Inverse Filtering (DIF), a spatially informed transformation designed to counteract spatial smoothing in functional-connectivity representations (i.e., functional networks) and thereby enhance their discriminative power for pattern recognition. Spatial smoothing in electroencephalography (EEG) signals and derived features, such as functional connectivity, is largely attributed to volume conduction. Functional connectivity has been increasingly used in brain–computer interface (BCI) studies; however, this spatial smoothing can introduce spurious connections and distort functional-connectivity patterns. **Methods:** DIF approximates spatial smoothing in functional connectivity, which is potentially associated with volume conduction, as a diffusion-like process and applies a regularized inverse operation to transform the observed functional networks into networks with enhanced discriminative representations. The effectiveness of DIF in enhancing the discriminative power of functional-connectivity representations in pattern recognition was evaluated using emotion recognition as the paradigm task, which is a critical component of BCI systems. Experimental results show that DIF generally improves emotion-recognition performance relative to the originally observed functional networks under electrode-sparsification conditions, with the most consistent improvements observed for Pearson correlation coefficient (PCC) estimation. Both signal-level processing, which operates on EEG signals before functional-connectivity estimation, and function-al-connectivity-level transformations, including graph signal processing (GSP)-based filtering applied after functional-connectivity estimation, were included as comparators. Under this framework, DIF is also considered a functional-connectivity-level transformation, but does not rely on a GSP framework. **Results:** The performance of DIF demonstrates the potential of functional-connectivity-level transformation as a complement or alternative to signal-level processing for enhancing connectivity-based pattern recognition. Overall, DIF improves classification performance and offers strong compatibility with modern functional-connectivity-based BCI pipelines.

## 1. Introduction

Electroencephalography (EEG)-based functional connectivity and the derived feature representation (typically functional networks) have been proposed for decades as features characterizing brain activity and have been increasingly used in brain pattern recognition, including disease diagnosis, motor imagery, and emotion recognition, among other brain–computer interface (BCI) applications.

Spatial smoothing in EEG and its derived features, such as functional connectivity, is considered to be largely caused by volume conduction. For functional connectivity, spatial smoothing is a classical yet critical source of artifact, generating spurious connections (i.e., inflated estimation) and distorting functional-network structures during connectivity estimation. Such spatial smoothing obscures discriminative structures and degrades both the performance and efficiency of downstream pattern-recognition tasks or BCI systems, highlighting the need for effective countermeasures.

### 1.1. BCI, EEG and Functional Connectivity

BCI, also known as brain-machine interface (BMI), has attracted considerable attention in both research and industry [1,2,3,4]. The vision of BCI is to establish a communication bridge between humans and computers. For example, one may construct a system that decodes a user’s intentions-such as motor movements through analysis of neural activity patterns [5,6]. Neural activity involves the generation of weak bioelectrical signals, which can be detected non-invasively using EEG [7,8,9]. With advances in neuroscience and machine learning, it is now possible to infer cognitive processes from the spatiotemporal patterns of these signals by combining signal processing, feature engineering, and machine learning techniques [5,6,10,11].

EEG is widely regarded as one of the most promising modalities for BCI because of its relatively low cost and portability [12,13]. A major advantage of EEG lies in its non-invasive nature; however, this same characteristic also means that the recorded signals are indirect and frequently contaminated by artifacts [14]. For example, major sources of such artifacts include volume conduction and the associated spatial smoothing [15,16].

Volume conduction arises from the conductive properties of head tissue, causing electrical signals to propagate across surrounding regions [15,16]. This spatial leakage reduces spatial specificity, introduces redundancy and source mixing into the recordings [17,18], and leads to a form of contamination that further propagate into the estimation of functional connectivity and the construction of derived functional networks, both of which serve as a critical foundation for feature engineering in connectivity-based BCI [16,19,20].

Functional connectivity is broadly defined as the statistical dependency or temporal association between neural signals recorded from different brain regions [16,20,21]. It characterizes temporal and spectral relationships among regions, thereby providing insights into patterns of neural interaction and information flow. 

The high temporal resolution of EEG has extended functional connectivity analysis into the dynamic domain, giving rise to the concept of dynamic functional connectivity. Dynamic functional connectivity and the dynamic functional networks derived from it capture rich temporal, structural, and topological features that have demonstrated effectiveness in brain pattern recognition tasks [22,23].

### 1.2. Volume Conduction

However, the estimation of functional connectivity is highly susceptible to artifacts induced by spatial smoothing, particularly those arising from volume conduction. 

Volume conduction represents a classical yet critical challenge in EEG-based systems, as the electrical fields recorded at the scalp surface are spatially blurred by the conductive properties of head tissues, resulting in spatially mixed signals and reduced spatial resolution. Furthermore, volume conduction not only blurs the underlying source distributions but also generates spurious connections that become embedded in functional networks during connectivity estimation [16,19,20].

A more concrete example arises when correlation is used to measure functional connectivity between two EEG signals. Volume conduction can cause two spatially adjacent but functionally unrelated channels to appear synchronous because they share spatially blurred activity originating from the same neural source. This artificially inflated synchrony, arising from volume conduction rather than genuine neural interactions, leads to the estimation of spurious connections and consequently introduces volume conduction-induced artifacts into functional connectivity. Such artifacts not only introduce false local features but can also collectively distort the overall structure of functional networks, ultimately compromising both the interpretability and discriminative power of the derived functional network patterns, particularly in tasks that require precise characterization of neural activity [16,19,20].

### 1.3. Related Work on Volume Conduction

To counteract volume conduction (or, more generally, spatial smoothing), conventional source localization techniques, such as minimum norm estimation (MNE), low-resolution brain electromagnetic tomography (LORETA) and its variants [24,25,26], attempt to mitigate this effect by reconstructing cortical sources from scalp potentials. However, their high computational complexity [27] and instability [28,29] render them unsuitable for real-time BCI applications.

ICA is a classical method for separating signal components, therefore it can also be used to suppress the volume conduction [30,31]. Recently, several studies have generalized ICA to the graph domain, introducing the potential for applying the method to functional connectivity-level analysis [32]. Nevertheless, ICA primarily aims to separate statistically independent source components rather than explicitly modeling or correcting volume conduction, and its performance depends heavily on assumptions regarding source independence and decomposition quality. More importantly, ICA results are often unstable across runs and sensitive to window partitioning settings, while also relying heavily on manual intervention. These limitations make ICA impractical for dynamic functional connectivity-based classification tasks, where manually identifying and removing artifact-susceptible components is infeasible and no reliable automated solution is currently available.

The current source density (CSD) transform is one of the most established and widely used approaches for counteracting the volume conduction [33,34]. By computing the second spatial derivative (surface Laplacian) of scalp potentials, CSD enhances spatial resolution and attenuates the spatial spread caused by volume conduction. However, CSD is fundamentally defined for EEG sensor signals and therefore operates at the signal level. Its formulation does not naturally extend to higher-level representations such as functional connectivity networks [35].

Modern deep learning frameworks treat functional networks as images, and this commonly used feature representation shifts our concentration toward image-based filtering. However, directly adapting image-based filtering to functional networks is non-trivial: image filters assume a regular grid, whereas EEG electrodes are irregularly spaced [34,36]. Consequently, the lack of an underlying grid to characterize EEG electrodes means the resulting structure loses meaningful spatial information.

To overcome the limitations of image-based filtering, graph-theoretical frameworks, particularly those derived from graph signal processing (GSP), have emerged as powerful tools for analyzing data on irregular domains [37,38]. Within this framework, graph spectral filtering (GSF) [39,40,41] and GSF using heat kernel (GSF-HK) [40,42,43,44] represent two classical paradigms that generalize spatial filtering to graph-structured data, enabling smoothness regulation and spectral diffusion analysis. Because volume conduction may contribute to spatially smooth patterns in functional connectivity representations, graph-based filtering provides a means of modifying such patterns according to inter-electrode geometry. Unlike conventional signal-level methods, such as CSD and SSD, which operate before connectivity estimation, GSF methods operate directly on functional connectivity representations after connectivity estimation.

### 1.4. Introduction of the Proposed Method

Existing approaches address volume conduction (or, more generally, spatial smoothing) either prior to functional connectivity estimation through signal- or source-level processing or after connectivity estimation by applying general GSP-based filters to graph representations. However, spatially informed inverse transformations formulated directly at the functional connectivity level, without relying on a GSP framework, remain unexplored. This gap motivates Diffusion Inverse Filtering (DIF), an alternative functional connectivity-level transformation based on a direct spatial diffusion model that counteracts spatial smoothing and thereby enhances functional connectivity representations.

DIF approximates spatial smoothing in functional networks, which is potentially associated with volume conduction, as a diffusion-like process and applies a regularized inverse operation to transform the observed functional networks into networks with enhanced discriminative representations.

**Figure 1:**
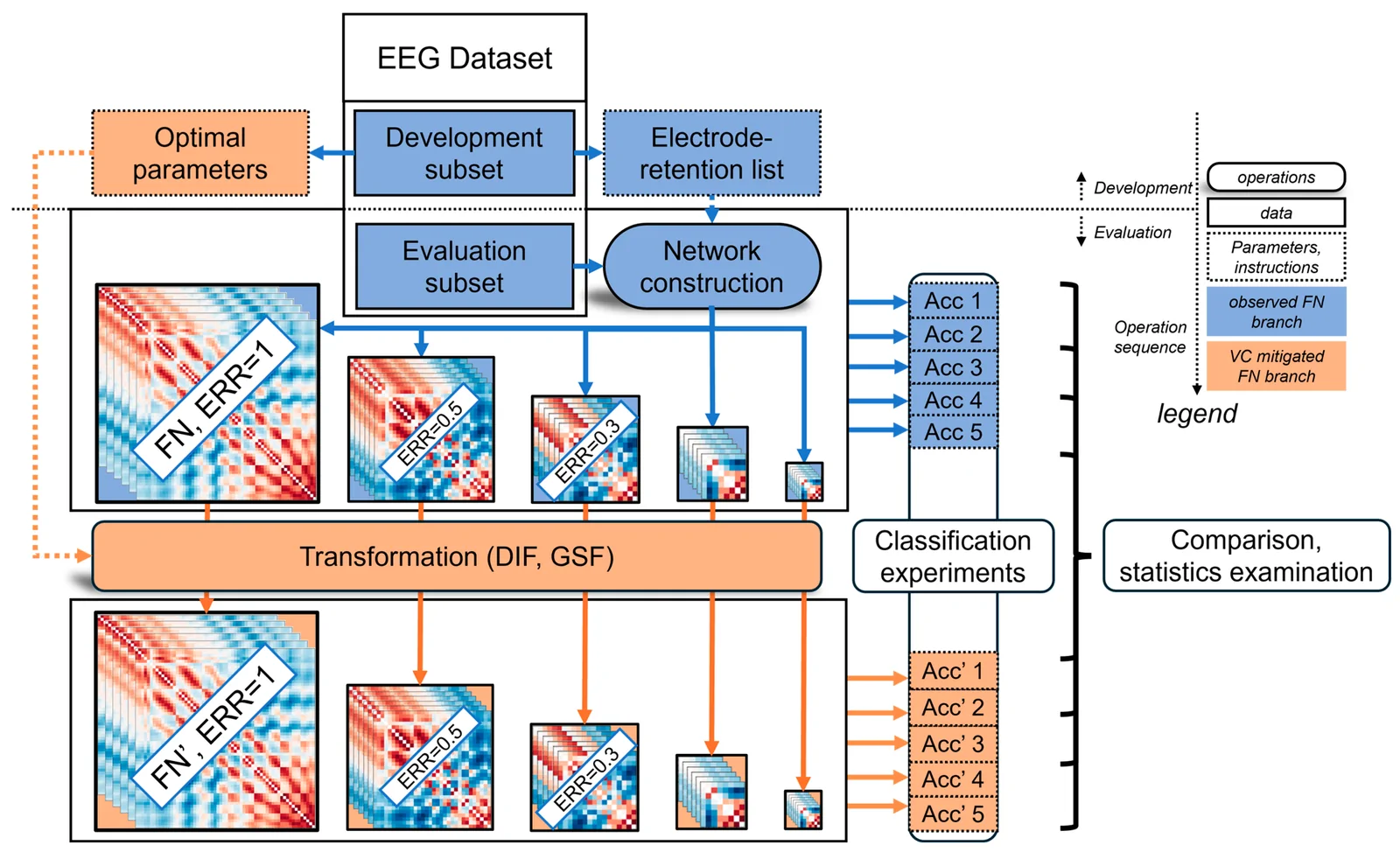
Illustration of the overall framework of this study, highlighting modeling, dataset partitioning, and the evaluation framework. Legend: *FN*: originally observed functional network; *FN*′: DIF-transformed functional-network representation; ERR: electrode-retention rate. Evaluations were conducted under various sparsification conditions. ERR determines the retained electrodes, thereby defining each sparsification condition. Description: The EEG dataset was divided into a development subset and an evaluation subset. Parameter optimization and determination of the electrode-retention list were performed on the development subset, whereas the evaluation subset was reserved for downstream classification experiments.

The proposed DIF and competing methods were evaluated by examining whether their transformed networks improved classification performance relative to the originally observed functional networks. The evaluation framework was constructed around an emotion-recognition task, which is an integral component of BCI systems. This framework is illustrated in Figure 1 and follows the evaluation rationale adopted in our previous work [45].

Experimental results show that DIF generally improves emotion-recognition performance relative to the originally observed functional networks under electrode-sparsification conditions and achieves performance comparable to that of the competing methods. These findings demonstrate its ability to enhance the discriminability of functional-connectivity representations.

## 2. Proposed Method

### 2.1. Framework

The proposed DIF is mathematically formalized by Equations (1)–(7). Rather than reproducing the complex biophysical propagation of electrical fields, DIF provides a simplified matrix-based model of spatial smoothing operating on functional networks. Motivated by the spatial spreading associated with volume conduction, DIF approximates this smoothing process as a bilateral diffusion:

(1) $$FN=K\;{FN}_{latent}K^{T},$$

where *FN* denotes the originally observed functional network obtained through standard functional-connectivity estimation, *FN_latent_* denotes a latent representation before the modeled spatial smoothing, and *K* is the spatial diffusion kernel. Here, *FN_latent_* is a modeling construct rather than a physiologically validated genuine functional network.

DIF then introduces a regularized inverse-filtering operation to counteract the assumed process:

(2) $$FN^{\prime }=K^{-1}FN\;{K^{-1}}^{T},$$

where *FN′* denotes the DIF-transformed functional network. Because of measurement noise, model simplification, and numerical limitations, *FN′* should not be interpreted as an exact reconstruction of *FN_latent_* or of the underlying physiological connectivity.

This formulation of (1) and (2) adopts bilateral diffusion, in which the kernel acts on both the row and column dimensions of the functional network. This symmetric construction reflects the pairwise structure of the matrix: each connection is jointly transformed according to the spatial neighborhoods of its two endpoints. The bilateral formulation therefore preserves matrix symmetry and provides a consistent basis for the subsequent inverse-filtering operation.

### 2.2. Diffusion Kernel

Gaussian filtering is widely used to model smoothing or blurring in data processing [46]. For data characterized by meaningful spatial distances, such as electrode-based functional-connectivity representations, the diffusion kernel can be defined as a Gaussian function of the inter-electrode distances:

(3) $$W_{i,j}=\exp\left(-\frac{{{DM}_{i,j}}^{2}}{2\sigma^{2}}\right),$$

where *DM* denotes the inter-electrode Euclidean distance matrix, *σ* controls the spatial scale of smoothing. To preserve the symmetry of the network and stabilize the filtering process, this weight matrix is symmetrically normalized using its degree matrix:

(4) $$G=D^{-\frac{1}{2}}W\;D^{-\frac{1}{2}},$$

where *D* is a diagonal degree matrix with entries $D_{ii}=\sum_{j}W_{ij}$. The resulting normalized Gaussian diffusion kernel *G* attenuates high-frequency spatial variations while preserving the intrinsic structural relationships encoded in the functional network.

### 2.3. Diffusion and Inverse Diffusion

After determining the diffusion kernel, DIF models the diffusion-like transformation and its inverse operation as:

(5) $$FN=G\;{FN}_{latent}G^{T},$$

(6) $$FN^{\prime }=G^{-1}FN\;{G^{-1}}^{T},$$

However, direct inversion of the Gaussian kernel *G* can be numerically unstable and ill-conditioned. To address this, the Tikhonov-regularized pseudo-inverse (*PI*) [47] is introduced to ensure stability when solving this ill-posed inverse problem:

(7) $$G^{-1}\approx PI={{(G}^{T}G+\lambda I)}^{-1}\cdot G^{T},$$

In pseudo-inverse computation, *λ* regulates the trade-off between numerical stability and reconstruction accuracy, with excessively small *λ* amplifying noise and excessively large *λ* over smoothing the filtered functional network.

### 2.4. Parameter Requirements

Equations (3) and (7) involve two key parameters: *σ*, which controls the spatial scale of diffusion through the Gaussian kernel, and *λ*, which acts as a regularization term to stabilize the pseudo-inversion. The selection of these parameters directly affects the filtering behavior, motivating the need for optimal parameter estimation.

## 3. Competing Methods

### 3.1. Current Source Density (CSD)

CSD is a signal-level spatial filtering technique widely used to reduce the effects of volume conduction in EEG by enhancing spatially local neural activity while attenuating broadly distributed components [36]. By approximating the second spatial derivative of the scalp potential, CSD improves spatial resolution and supports more reliable estimation of functional connectivity.

In this study, CSD was computed using the spherical spline interpolation method proposed by Perrin et al. [48], which estimates the scalp current source density directly from electrode coordinates distributed on a unit sphere. Unlike finite-difference approaches, the spherical spline method computes the surface Laplacian analytically from a continuous spline representation of the scalp potential without explicitly defining local neighborhoods, resulting in improved numerical stability and applicability to arbitrary electrode configurations [48,49].

Former studies have discussed the determination of these parameters. Across most literature, *m* ranges from 2 to 5, and *λ* ranges from 10^−2^ to 10^−9^. However, a combination of *m* = 4 and *λ* = 10^−5^ is recommended, as it serves as a robust constant for a wide range of EEG applications [50]; this configuration is also adopted as the default setting for the CSD Toolbox [51]. Follows the recommendation, this recommended combination of settings was applied in the current study.

For comparison with other methods involved in this study, in the experimental branch for CSD, the CSD was applied once to the broadband EEG signals before frequency decomposition. The resulting CSD signals (EEG signals altered by CSD) were subsequently band-pass filtered into the corresponding frequency bands (delta, theta, alpha, beta and gamma), and all functional connectivity analyses were performed using the filtered CSD signals instead of the filtered EEG signals.

### 3.2. Spatial Spectral Decomposition (SSD)

SSD is a signal-level spatial filtering method that enhances oscillatory activity in a target frequency band by maximizing its variance relative to neighboring frequency bands [52,53]. Although SSD is not explicitly designed to suppress volume conduction, its ability to improve the signal-to-noise ratio makes it a valuable baseline for comparison with the proposed method. SSD is formulated as a generalized eigenvalue problem:

(8) $$C_{s}w=\lambda C_{n}w.$$

where *C_s_* and *C_n_* denote the covariance matrices of the target frequency band and the neighboring frequency bands, respectively. And *λ* is the generalized eigenvalue representing the ratio of signal variance to neighboring-band variance. The eigenvectors corresponding to the largest eigenvalues form the spatial filters, which are applied to the multichannel EEG signals to obtain band-enhanced components.

For comparison with the other methods, SSD was implemented using zero-phase Butterworth filters to define the target and neighboring frequency bands. The six components associated with the largest generalized eigenvalues were retained for each frequency band, thereby preserving oscillatory activity with the highest spectral SNR while discarding components dominated by neighboring-band activity.

SSD replaced the conventional band-pass filtering (delta, theta, alpha, beta, and gamma), and all subsequent functional connectivity analyses were performed on the EEG signals reconstructed from the retained SSD components rather than directly from band-pass filtered EEG signals.

### 3.3. First-Order Graph Spectral Filtering (GSF-FO)

Compared with signal-level processing methods, such as CSD and SSD, GSP provides an alternative approach that operates directly on functional-connectivity representations, such as functional networks. GSP has been widely adopted in EEG analysis [54,55] and brain-imaging applications involving functional connectivity [37,56,57], based on established GSP theoretical frameworks [40,58,59].

GSF-FO employs a first-order polynomial graph spectral filter to modify low-frequency spatial components in functional networks. These components may reflect spatially smooth patterns influenced by volume conduction, although they cannot be attributed exclusively to it. Rather than operating on EEG signals, GSF-FO performs filtering directly on graph signals defined over the functional-network vertices [37,38,41].

Based on the inter-electrode distance matrix *DM*, a Gaussian function is used to construct the weighted adjacent matrix:

(9) $$W_{i,j}=\exp\left(-\frac{{{DM}_{i,j}}^{2}}{{2\sigma }^{2}}\right),W_{i,i}=0,$$

the corresponding degree matrix and the normalized graph Laplacian are defined as:

(10) $$D_{i,i}=\sum_{j}W_{i,j},L=I{-D}^{-\frac{1}{2}}W\;D^{-\frac{1}{2}},$$

Following the GSP framework, graph filtering is formulated as a function of the graph Laplacian,

(11) F=h(L),

where *h*(⋅) denotes the graph spectral response. Here, a first-order polynomial graph filter is adopted, yielding

(12) $$F=h(L)=\left\{\begin{array}{c} I-\alpha L,\;\mathrm{low}\;\mathrm{pass},\\ \;\;\;\;\;\;\;\alpha L,\;\mathrm{high}\;\mathrm{pass}, \end{array}\right.$$

where *α* controls the filtering strength. Although implemented directly as a polynomial of the graph Laplacian without explicit eigendecomposition, this formulation is mathematically equivalent to graph spectral filtering because the polynomial response corresponds to the spectral response *h*(*λ*) in the graph Fourier domain. The filtering is then defined as:

(13) $${FN}^{\prime }=F\;FN\;F^{T},$$

where *FN* denotes the originally observed functional network and *FN*′ denotes the GSF-FO-transformed functional-network representation.

The bilateral filtering formulation is consistent with the bilateral formulation adopted in DIF, similarly approximating propagation across both row and column dimensions. The low-pass filter attenuates high-frequency graph components while retaining spatially smooth patterns. Conversely, the high-pass filter attenuates low-frequency components and emphasizes localized spatial variations. The high-pass variant is adopted in this study to reduce the dominance of spatially smooth patterns that may be influenced by volume conduction.

For simplicity of model structure, the scaling factor is fixed at *α* = 1. Thus, once the inter-electrode distance matrix *DM* is specified, the only free parameter in the model is the Gaussian kernel width *σ*, which controls the spatial scale of the graph construction and determines the neighborhood sensitivity of the filtering operation.

### 3.4. Heat Kernel Graph Spectral Filtering (GSF-HK)

To serve as a secondary GSP competitor, this method employs a heat kernel graph spectral filter based on the explicit eigendecomposition of the graph Laplacian [58]. As in GSF-FO, the functional network is regarded as a graph, while filtering is performed explicitly in the graph spectral domain.

For GSF-HK, the construction of the weighted adjacency matrix, the corresponding degree matrix, and the normalized graph Laplacian follows the exact procedure detailed for GSF-FO in Equations (9) and (10).

Unlike GSF-FO, which directly implements a polynomial of the graph Laplacian, GSF-HK explicitly performs the eigendecomposition *L* = *UΛU^T^*, where *U* = [*u*_1_, …, *u_N_*] ∈ *ℝ ^N^*^×*N*^ is the orthonormal graph Fourier basis and *Λ = diag*(*λ*_1_, …, *λ_N_*) with 0 = *λ*_1_ ≤ *λ*_2_ ≤ …≤*λ_N_*.

Following the GSP framework, graph filtering is expressed through the spectral response:

(14) $$F=h\left(L\right)=U\;h\left(\Lambda \right)\;U^{T},$$

where a heat kernel response is adopted to define *h*(⋅). The heat kernel response are formulated as:

(15) $$h\left(\lambda \right)=\left\{\begin{array}{c} \;\;\;\;\exp(-t\lambda ),\;\;\mathrm{low}\;\mathrm{pass}\\ 1-\exp(-t\lambda ),\;\;\mathrm{high}\;\mathrm{pass} \end{array}\right.,t>0,$$

the filtered spectral matrix is then constructed as:

(16) $$H=h\left(\Lambda \right)=diag\left(h\left(\lambda_{1}\right),\ldots ,h\left(\lambda_{N}\right)\right),$$

and the graph filter is given by:

(17) $$F=U\;H\;U^{T}.$$

Consistent with the bilateral formulation adopted in DIF and GSF-FO, the filter acts on both the row and column dimensions of the functional network. The resulting GSF-HK-transformed representation is obtained as:

(18) $${FN}^{\prime }=F\;FN\;F^{T}.$$

For simplicity of model structure, the filtering strength is fixed at *t* = 1. Thus, once the inter-electrode distance matrix *DM* is specified, the only free parameter in the model is the Gaussian kernel width *σ*.

The main difference between GSF-FO and GSF-HK lies in their spectral responses. GSF-FO employs a first-order polynomial response with linear frequency weighting, whereas GSF-HK adopts the exponential response [42,43,44], providing smooth and continuous spectral attenuation. Consequently, GSF-HK generally offers more gradual filtering and greater robustness to noise in EEG functional networks.

## 4. Dataset and Feature Engineering

### 4.1. SEED Dataset and Preprocessing

The method evaluation for brain pattern recognition was conducted using the SJTU Emotion EEG Dataset (SEED) [60], a widely adopted benchmark for emotion recognition studies [61]. The dataset comprises 62-channel 200 Hz EEG experimental recordings collected according to the international 10–20 system from fifteen subjects, each of whom participated in three independent experiments, resulting in 45 EEG recordings of the same length in total.

In each experiment, subjects viewed fifteen pre-labeled video clips (approximately four minutes per clip, total 3394 s) designed to elicit emotions across three valence categories: negative, neutral, and positive. Each trial in the recordings was annotated with the corresponding valence labels. Consequently, the dataset used in this study consists of 45 EEG recordings, each with 62 channels, a sampling rate of 200 Hz, and a duration of 3394 s, together with the corresponding emotional valence annotations.

The preprocessing followed the SEED developers. The data were then processed using a band-pass filter from 0.3 Hz to 50 Hz to reduce noise and artifacts.

The preprocessed EEG signals were filtered into five classical frequency bands: delta (1–4 Hz), theta (4–8 Hz), alpha (8–13 Hz), beta (13–30 Hz), and gamma (30–45 Hz). Among these, the alpha, beta, and gamma bands were selected for feature extraction, as higher-frequency activity is more strongly associated with emotional processing [60].

### 4.2. DREAMER Dataset and Preprocessing

The method evaluation was further conducted using the DREAMER dataset [62], a multimodal database widely used for emotion recognition based on physiological signals. The dataset contains EEG and ECG recordings from 23 subjects exposed to emotion-eliciting audiovisual stimuli (total 3729 s), along with self-reported emotion labels. EEG signals were recorded from 14 electrodes positioned according to the international 10–20 system at a sampling rate of 128 Hz.

Each subject viewed 18 film clips designed to elicit different emotional responses, resulting in 18 EEG trials per subject and 414 trials in total. After each trial, participants rated their emotional states in terms of valence, arousal, and dominance on a five-point scale. Each EEG trial was therefore associated with corresponding valence, arousal, and dominance labels. In this study, only the valence labels were used and transformed to three-point scale representing positive, neutral and negative emotional states (1, 01 -1).

The preprocessing followed the DREAMER developers. The data were then processed using a band-pass filter from 0.3 Hz to 50 Hz to reduce noise and artifacts. Consistent with the SEED dataset, the preprocessed EEG signals were filtered into the same classical frequency bands.

### 4.3. Estimation of Functional Connectivity

Functional brain networks can be constructed using various connectivity measures, such as the Pearson correlation coefficient (PCC), phase-locking value (PLV), phase-lag index, mutual information, and others [20]. In this study, PCC and PLV were employed because of their widespread use and computational simplicity. Both measures are sensitive to spatial smoothing, making them suitable for examining how DIF transforms amplitude- and phase-based functional-connectivity representations.

For time series such as EEG signals *x_ci_* and *x_cj_*, both with *T* sampling points, PCC quantifies the linear relationship between two signals, which is defined as:

(19) $$PCC\left(x_{ci},x_{cj}\right)=\frac{\sum_{t=1}^{T}\left(x_{ci,t}-{\bar{x}}_{ci}\right)\left(x_{cj,t}-{\bar{x}}_{cj}\right)}{\sqrt{\sum_{t=1}^{T}{\left(x_{ci,t}-{\bar{x}}_{ci}\right)}^{2}}\sqrt{\sum_{t=1}^{T}{\left(x_{cj,t}-{\bar{x}}_{cj}\right)}^{2}}},$$

where $\bar{x_{ci}},\bar{x_{cj}}$ are the respective means.

The PLV measures the phase synchronization strength between two signals across time:

(20) $$PLV(x_{ci},x_{cj})=|\frac{1}{T}\sum_{t=1}^{T}e^{j(\phi_{x_{ci}}(t)-\phi_{x_{cj}}(t))}|,$$

where $\phi_{x_{ci}}(t)$ and $\phi_{x_{cj}}(t)$ are the instantaneous phases of the two signals, $j=\sqrt{-1}$.

The PCC value ranges from −1 (perfect negative correlation) to 0 (no linear relationship) to 1 (perfect positive correlation). In contrast, the PLV value ranges from 0 (no synchronization) to 1 (perfect phase locking).

Except for connectivity visualization, where the diagonal entries were set to NaN, no manual operations were applied to the diagonal elements throughout the study, including during functional connectivity estimation, DIF, GSF, or prior to inputting the networks into the classification models. This is to preserve the mathematical assumptions underlying DIF and GSF and to avoid introducing potential bias in the comparison across methods.

### 4.4. Construction of Functional Networks

To enable the downstream classification task, dynamic functional networks were constructed following the established dynamic functional connectivity framework.

The preprocessed EEG signals were segmented into non-overlapping 1 s windows, yielding *N* windows with *T* data points per window. Accordingly, the EEG data for each experiment were represented as an *N* × *T* × *C* tensor, where *N* (3394 for SEED, 3729 for DREAMER) denotes the number of 1 s windows, and *C* (62 for SEED, 14 for DREAMER) denotes the number of EEG channels.

Functional connectivity was then estimated for each segmented EEG window and separately for each frequency band, resulting in a collection of functional networks with dimensions *N* × *B* × *C* × *C* for each experiment, where *B* = 3 represents the three frequency bands (alpha, beta, and gamma).

In the downstream classification task, each functional network tensor (shape of *N* × *B* × *C* × *C*) served as the input to a single classification experiment.

Across the SEED dataset, each of the 15 subjects participated in three independent EEG recording sessions, resulting in three functional network collections per subject and 45 collections in total. In the DREAMER dataset, each of the 23 subjects participated in one EEG recording session, resulting in one functional network collection per subject and 23 collections in total.

### 4.5. Construction of Functional Networks Under Sparsification Conditions

Comparisons across methods based solely on functional networks constructed from the full set of EEG electrodes may not fully reveal performance differences, as redundant or weakly informative connections can obscure variations in the density of discriminative information, causing all methods to achieve similarly high classification performance.

To enable a more rigorous comparison, the methods were further evaluated under different sparsification conditions—specifically, at reduced electrode densities. For each sparsification condition, functional networks were constructed using a reduced set of EEG electrodes corresponding to the target electrode density. The methods were then evaluated on these reduced-electrode functional networks to assess their robustness and effectiveness under varying electrode densities.

The electrode sparsification conditions were defined by different electrode-retention rates (ERRs), which represent the proportion of electrodes retained from the original electrode set. Specifically, electrode sparsification was determined by an electrode-retention list, in which electrodes were ranked according to their importance. For a given ERR (ranging from 0 to 1, or equivalently 0% to 100%), the top-ranked electrodes were retained to construct the corresponding reduced-electrode functional networks. Consequently, each functional network had dimensions of *N* × *B* × *C_ERR_* × *C_ERR_*, where *C_ERR_* denotes the number of retained electrodes under the specified ERR.

The importance of each electrode was quantified by its average node strength, computed over all windows and frequency bands from the originally observed functional networks:

(21) $$S_{i}=\frac{1}{B\times N\times C}\sum_{b=1}^{B}\sum_{n=1}^{N}\sum_{j=1}^{C}{FN}_{i,j}^{n,b}.$$

To ensure a fair comparison among the methods, a unified electrode-retention list (*S_i_*) was generated from the originally observed functional networks and applied consistently to DIF, GSF-FO, and GSF-HK. Alternatively, adaptive, method-specific electrode-retention lists (*S’_i_*) can be obtained by computing electrode importance from the corresponding transformed functional-network representations:

(22) $${S^{\prime }}_{i}=\frac{1}{B\times N\times C}\sum_{b=1}^{B}\sum_{n=1}^{N}\sum_{j=1}^{C}{{FN}^{\prime }}_{i,j}^{n,b}.$$

CSD and SSD differ from the above methods in that they are more appropriately regarded as EEG preprocessing methods rather than methods operating directly on the observed functional networks. Because the functional networks for CSD and SSD were constructed only after applying the corresponding preprocessing procedure, the unified electrode-retention list derived from the original observed functional networks was not directly applicable to them. Therefore, for the observed, CSD, and SSD functional networks, the electrode-retention list used in the unified-retention analysis was identical to that used in the adaptive-retention analysis, because in each case the list should be derived from the corresponding network itself. Specifically, list(observed) = list′(observed), list(CSD) = list′(CSD), and list(SSD) = list′(SSD). However, because these lists were derived independently from different functional networks, they were not necessarily identical across the original, CSD, and SSD networks, i.e., list(observed) ≠ list(CSD) ≠ list(SSD).

Although the unified electrode-retention list provides a fair basis for comparison, adaptive electrode-retention lists may further reveal the potential of individual methods by allowing the retained electrodes to better reflect the characteristics of the corresponding processed networks.

## 5. Evaluation Framework

The proposed DIF and competing methods were evaluated by examining whether their transformed functional-connectivity representations (i.e., functional networks) improved performance in offline connectivity-based pattern recognition. Emotion recognition, an integral paradigm in BCI research, was adopted as the evaluation task.

The evaluation framework includes data partitioning, parameter optimization, functional network construction under sparsification conditions, and independent classification experiments for emotion recognition under sparsification conditions for each method.

The protocol for using the dataset is to divide it into two separate subsets: the development subset (5 subjects, total 15 recordings for SEED, 8 subjects, total 8 recordings for DREAMER) and the evaluation subset (10 subjects, total 30 recordings for SEED, 15 subjects, total 15 recordings for DREAMER). The development subset was used exclusively for parameter optimization and calculation of the electrode importance for determining the sparsification conditions. The evaluation subset was used exclusively for classification experiments within the evaluation framework. The methods were primarily evaluated on the SEED dataset, while the DREAMER dataset was used for supplementary external validation to assess cross-dataset generalizability.

SOTA models and scores were not the primary focus of this study. Instead, a minimalist convolutional neural network (CNN; Table 1) was designed for the emotion classification experiments to ensure training stability, reduce confounding variables, and minimize the risk of architectural bias.

In each independent classification experiment, a single collection of functional networks (*N* × *B* × *C* × *C)* was used as the input. This is also known as an experiment-dependent or intra-experiment paradigm. A 5-fold cross-validation scheme was adopted following the common practice in EEG-based emotion recognition studies [63,64,65,66].

A three-category classification scheme was applied to both the SEED and DREAMER datasets. For the DREAMER dataset, the original labels were approximated into three classes—positive, neutral, and negative—corresponding to 1, 0, and −1, respectively.

The network was optimized using the Adam optimizer with an initial learning rate of 5 × 10^−4^ and a batch size of 128 for 30 epochs. The cross-entropy loss was adopted as the optimization objective. No learning-rate scheduling or early stopping strategy was employed. The random seed used for model initialization was set to 42. The final performance was reported as the average across the five folds. To preserve the temporal order of the samples and reduce the risk of data leakage between adjacent windows, no shuffling was performed prior to fold partitioning, while a stratification strategy was applied. As an additional control analysis, performance variability under shuffled conditions was also evaluated.

## 6. Parameter Optimization

The parameter optimization was conducted on the development subset. The results of parameter settings are shown in Table 2. All methods (DIF, GSF-FO, and GSF-HK) employ a Gaussian kernel, where *σ* controls the spatial diffusion extent, while DIF additionally introduces a regularization parameter *λ*.

For GSF-FO and GSF-HK, the high-pass variant is adopted to attenuate smooth, low-frequency graph components that may be influenced by volume conduction.

### 6.1. Unified Selection of σ

The scale parameter *σ*, which defines the spatial footprint of diffusion, is estimated once and shared across all methods to ensure a fair comparison under a common setting. After normalizing the inter-electrode distances to [0, 1], *σ* is computed as the median of all k-nearest-neighbor inter-electrode distances with *k* = 3. This choice reflects the typical local neighborhood of each electrode in the 10–20/10–10 EEG layout, where the three nearest electrodes provide a representative estimate of the characteristic local spacing while avoiding sensitivity to isolated electrode pairs or more distant neighbors. Consequently, the resulting σ captures the local spatial scale associated with volume conduction rather than a broader global topology. Using the median over all electrodes further improves robustness by reducing the influence of local geometric irregularities in electrode placement.

### 6.2. Determination of λ

For DIF, once *σ* is fixed, the remaining free parameter *λ* is determined using the L-curve criterion.

The L-curve implementation follows the standard formulation in Per Christian Hansen’s regularization theory [67]. Specifically, a logarithmically spaced grid of candidate *λ* values is evaluated within the same diffusion-inverse formulation as in (7). For each *λ*, the Tikhonov-regularized inverse is constructed, and both the residual norm and the regularization norm are computed. The optimal *λ* is then selected as the point of maximum curvature on the L-curve, corresponding to the conventional and widely adopted criteria.

To prevent data leakage and ensure a fair comparison, the L-curve optimization is restricted to a development subset. Specifically, the optimization is performed on the globally averaged functional network across the subset, with ERR equal to 100%.

After determining the parameters *σ* and *λ*, they are kept fixed for all reduced-electrode functional networks, as the underlying diffusion process is assumed to remain unchanged across different electrode densities, while using fixed parameters also avoids repeated optimization and ensures a fair comparison across reduced-network settings.

### 6.3. Stability and Effectiveness of Parameter Selection

**Figure 2:**
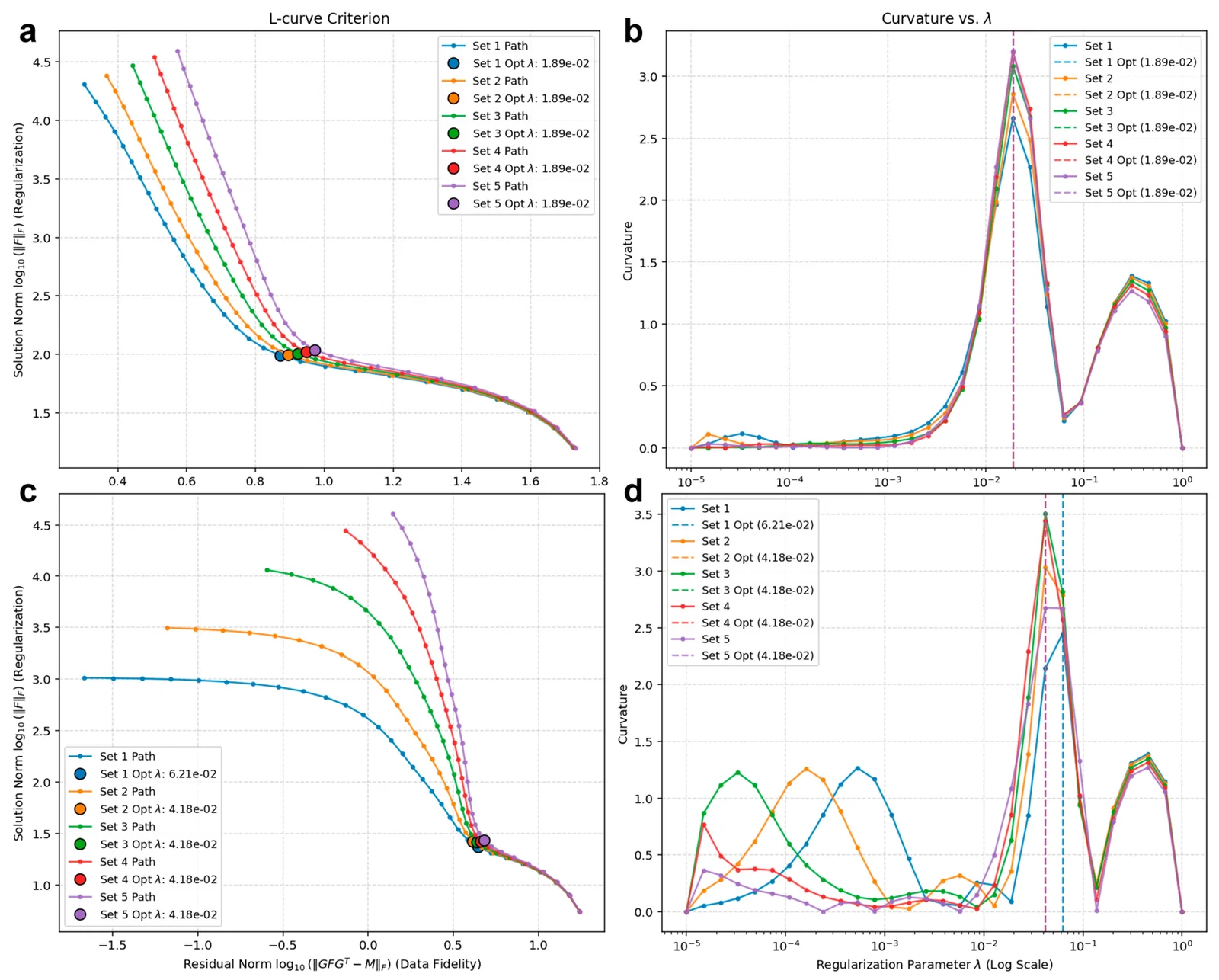
L-curve diagnostics across different settings of *σ* (determined by *k*-NN with *k* = 1 to 5, corresponding to Set 1 to Set 5) for optimal regularization parameter *λ* selection (**a**,**b**): SEED; (**c**,**d**): DREAMER). (**a**,**c**) The classical L-curve, representing the trade-off between the residual norm (data fidelity, x-axis) and the solution norm (regularization constraint, y-axis) in log-scale. The red circle highlights the selected optimal parameter (*λ* = 0.0189 for SEED; *λ* = 0.0621 for DREAMER with *k* = 1; and *λ* = 0.0418 for DREAMER with *λ* = 2, 3, 4, 5). (**b**,**d**) Discrete curvature of the L-curve plotted against the regularization parameter *λ* (log-scale). The dashed red vertical line locates the maximum curvature point, corresponding directly to the optimal *λ*.

To further evaluate the robustness of the proposed parameter selection strategy, the L-curve analysis was first repeated using five different values of the diffusion scale parameter σ, determined from the k-nearest-neighbor (*k*-NN) graph with *k* = 1 to 5 (Figure 2). Although different values of k slightly changed the spatial diffusion scale, the resulting L-curves exhibited highly consistent shapes (Figure 2a,c). More importantly, the corresponding curvature curves (Figure 2b,d) all reached their maximum at their respective optimal regularization parameters (*λ* = 0.0189 for SEED; *λ* = 0.0621 for DREAMER with *k* = 1; and *λ* = 0.0418 for DREAMER with *λ* = 2, 3, 4, 5). This consistency indicates that the selected regularization parameter is largely insensitive to moderate variations in the diffusion kernel, demonstrating the robustness of the L-curve criterion with respect to the construction of the graph Laplacian.

**Figure 3:**
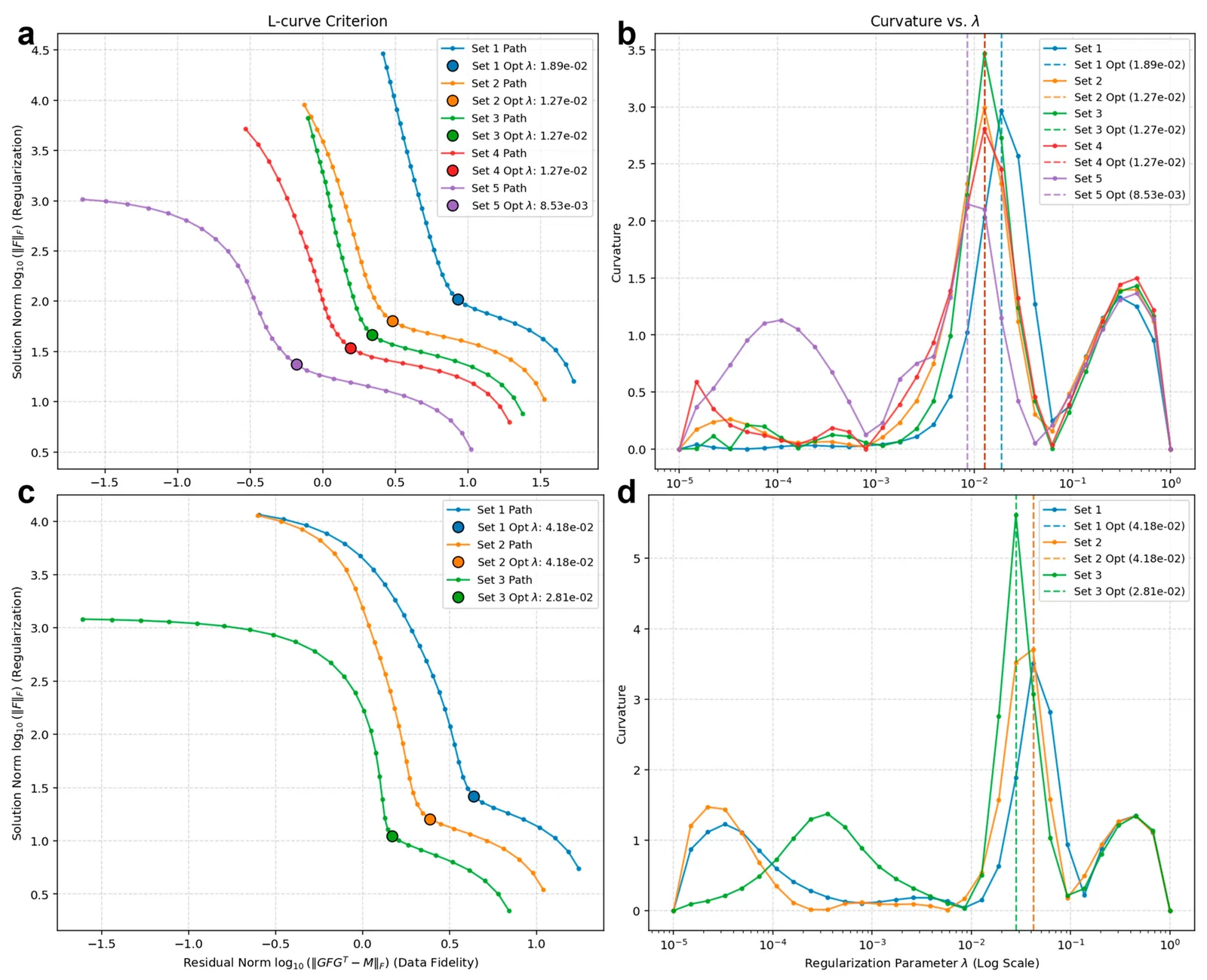
L-curve diagnostics across different settings of *σ* for selecting the optimal regularization parameter *λ* (**a**,**b**): SEED; (**c**,**d**): DREAMER). The *σ* settings were determined using ERR = {1, 0.5, 0.3, 0.2, 0.1}, corresponding to Sets 1 to Set 5 for SEED, and ERR = {1, 0.5, 0.3}, corresponding to Sets 1 to Set 3 for DREAMER.

To further examine the robustness of parameter selection across different reduced-electrode configurations (Figure 3), the retained electrodes were determined using ERR values of {1, 0.5, 0.3, 0.2, 0.1} for SEED and {1, 0.5, 0.3} for DREAMER, while fixing *k* in *k*-NN at 3.

As expected, reducing the number of electrodes altered both the residual norm and the solution norm, resulting in noticeable shifts in the L-curve trajectories (Figure 3a,c). Nevertheless, the characteristic L-shaped corner remained well preserved for all electrode montages. Correspondingly, the curvature curves (Figure 3b,d) consistently exhibited a distinct dominant peak within a narrow range of regularization parameters. Despite substantial reductions in electrode density, the resulting optimal values varied only slightly, ranging from 8.53 × 10^−3^ to 1.89 × 10^−2^ for SEED and from 2.81 × 10^−2^ to 4.18 × 10^−2^ for DREAMER.

These observations suggest that the inverse diffusion formulation preserves a stable balance between data fidelity and regularization across different graph constructions and reduced-electrode configurations. Therefore, selecting a single fixed regularization parameter from the development subset represents a reasonable compromise, supporting its use throughout the subsequent experiments while avoiding repeated parameter optimization.

It should be noted that the parameter optimization, including the L-curve analysis, was performed on the globally averaged functional network constructed from the development subset. Averaging across recordings substantially reduces random fluctuations and produces a smoother optimization landscape, which may contribute to the highly consistent L-curve characteristics observed in Figure 2 and Figure 3. In practical applications, however, DIF is applied to dynamic functional networks estimated from individual time windows. Under these conditions, the inverse diffusion operation may amplify measurement noise and numerical errors, resulting in less stable L-curve characteristics than those observed during parameter optimization. Nevertheless, because the parameter optimization is intended to determine a single global operating point rather than adapt to each individual network, the use of the averaged functional network provides a robust and practical strategy.

## 7. Evaluation Results

### 7.1. Classification Performance (SEED)

For SEED dataset, each recording from the evaluation subset (10 subjects, 30 recordings in total) was treated as an independent experiment, resulting in 30 experiments. All reported average classification scores, error estimates, and statistic evaluations were based on these 30 independent experiments (*n* = 30).

**Figure 4:**
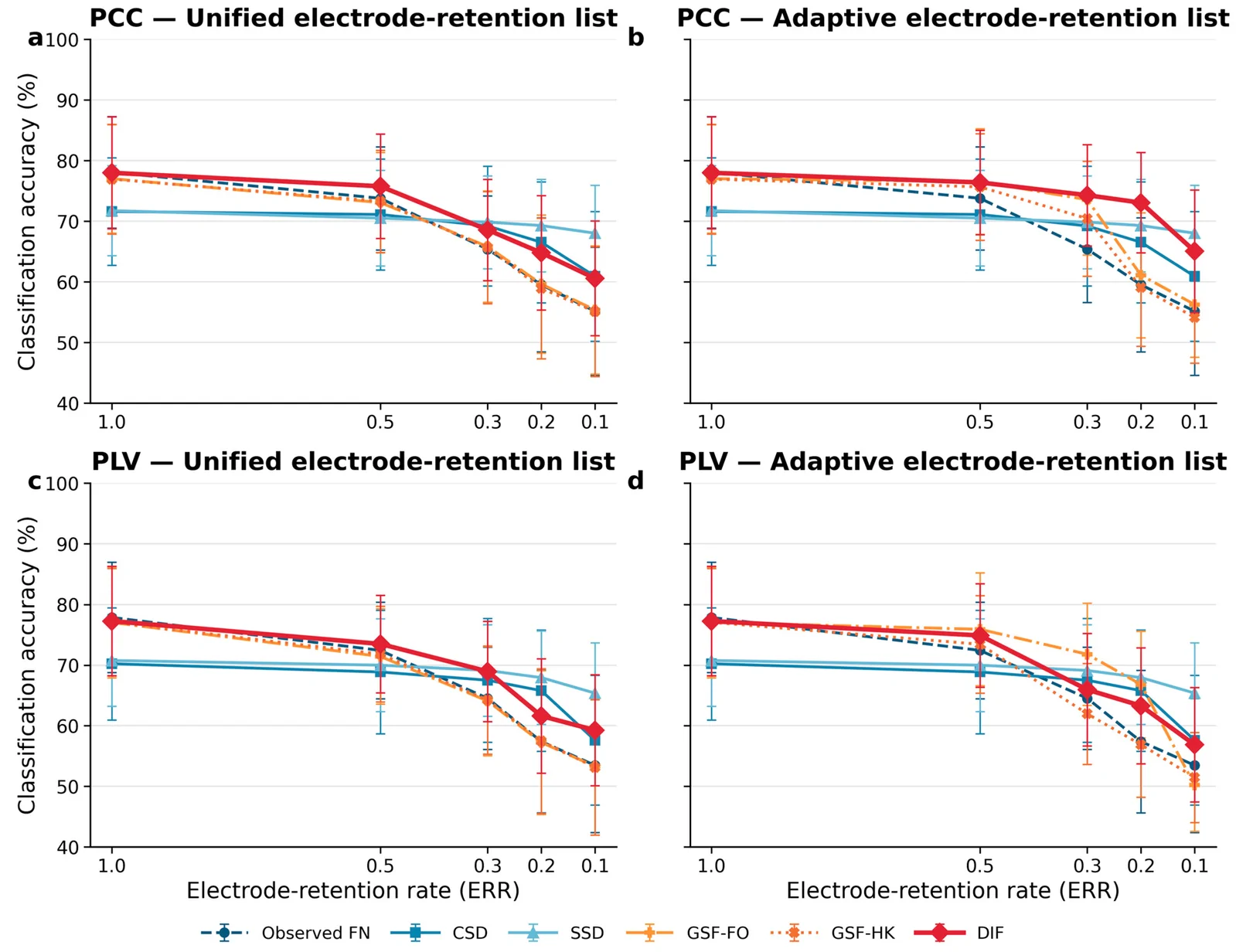
Average classification accuracy (*n* = 30, error bars: 95% CI) achieved by the different methods across varying ERRs, functional connectivity metrics, and electrode-retention strategies. (**a**) PCC with a unified electrode-retention list. (**b**) PCC with an adaptive electrode-retention list. (**c**) PLV with a unified electrode-retention list. (**d**) PLV with an adaptive electrode -retention list.

As shown in Figure 4, the proposed DIF was compared with the baseline (the original observed functional networks), two signal-level competitors (CSD and SSD), and two functional-connectivity-level competitors (GSF-FO and GSF-HK). Reduced-electrode functional networks were constructed at ERRs of {1, 0.5, 0.3, 0.2, 0.1}, corresponding to network sizes of 62 × 62, 31 × 31, 19 × 19, 13 × 13, and 7 × 7, respectively, for the 62-channel EEG dataset.

Overall, the most prominent trend across all four subplots is the negative correlation between electrode sparsity and classification accuracy. 

Under higher electrode densities (ERR 1.0 to 0.5), all evaluated methods maintain robust and relatively stable performance, generally hovering between 70% and 78% accuracy. The removal of up to 50% of the electrodes (ERR = 0.5) induces only marginal degradation in classification performance.

Moderate to lower electrode densities (ERR 0.3 to 0.1), a large decline in accuracy is observed across all methods as the ERR drops below 0.3. The most precipitous drop occurs between ERR 0.2 and 0.1, where accuracies fall to between 50% and 68%.

Comparison of electrode-retention strategies, comparing the left column (Unified list) with the right column (Adaptive list) reveals that the Adaptive electrode-retention strategy generally provides greater resilience against performance degradation at moderate sparsity levels (ERR 0.3 and 0.2). Methods utilizing the adaptive strategy maintain a tighter grouping and slightly higher average accuracies before the sharp decline at ERR 0.1. Both PCC and PLV metrics exhibit similar overall behavioral curves under these strategies.

#### 7.1.1. Method-Specific Performance (SEED)

An analysis of the individual algorithms reveals distinct performance characteristics across the ERR spectrum and further clarifies the relative advantages of the proposed framework.

Observed FN (Dark Blue Circle—Baseline): The originally observed functional network serves as the baseline for all evaluated methods. Its classification accuracy generally decreases as the ERR is reduced, with a more pronounced deterioration emerging below ERR = 0.5 compared with competitors, particularly under the adaptive electrode-retention setting. Nevertheless, the baseline is not uniformly the worst-performing representation: at higher electrode densities (ERR = 1.0, 0.5), its performance remains comparable to several methods and superior to signal-level methods (CSD, SSD).

DIF (Red Diamond—Proposed Method): The DIF-transformed functional-connectivity representations generally achieved favorable classification performance at full and moderate electrode-retention rates. At ERR = 1.0, classification accuracy reached approximately 77–78% across the four evaluated configurations, placing DIF among the highest-performing methods. Its advantage became more apparent at ERR = 0.5, where DIF achieved classification accuracies comparable to or higher than those of the competing methods. For PCC-based representations, this favorable performance generally remained evident at ERR = 0.3. Across ERR = 1.0, 0.5, and 0.3, DIF consistently improved classification performance relative to the original untransformed baseline and generally outperformed the signal-level methods.

Under more substantial electrode reduction, the classification performance of the DIF-transformed representations also decreased. Although DIF continued to improve upon the baseline under many conditions, the signal-level methods showed greater resilience at lower electrode densities and achieved higher classification accuracies in several cases at ERR = 0.2 and 0.1. At ERR = 0.1, DIF achieved approximately 61% and 65% accuracy for PCC under the unified and adaptive electrode-retention settings, respectively, and approximately 59% and 57% for PLV.

The relative behavior of the functional-connectivity-level and signal-level methods differed across the ERR range. Compared with the functional-connectivity-level competitors, DIF achieved higher classification accuracy under most evaluated conditions; the principal exception occurred for PLV with adaptive electrode retention, where GSF-HK achieved higher accuracy at ERR = 0.3 and 0.2. Compared with the signal-level methods, DIF generally achieved higher classification accuracy at ERR = 1.0 and 0.5 and remained broadly comparable at ERR = 0.3. At lower electrode densities, particularly ERR = 0.2 and 0.1, CSD and SSD showed greater robustness to electrode reduction and exceeded DIF under several conditions.

A representative result was observed for PCC under the adaptive electrode-retention setting, where the DIF-transformed representation achieved the highest or near-highest classification accuracy across almost the entire ERR range.

CSD (Steel Blue Square): The functional-connectivity representations obtained after CSD generally showed relatively stable classification performance across the evaluated electrode-retention rates. Although their classification accuracy was often lower than that of several other representations at high and moderate electrode densities, the reduction in performance with decreasing ERR was comparatively gradual. As a result, the CSD-based representations remained competitive with, and under some conditions exceeded, several functional-connectivity-level representations at lower electrode densities.

SSD (Light Blue Triangle): The functional-connectivity representations obtained after SSD showed comparatively limited performance degradation under substantial electrode reduction. Their classification accuracy changed more moderately with decreasing ERR than that of most other evaluated representations. At ERR = 0.1, the SSD-based representations achieved the highest classification accuracy across the four evaluated configurations, with values of approximately 65–68%. This pattern suggests that the relatively lower performance observed at higher electrode densities was accompanied by greater stability under severe electrode reduction.

GSF-FO and GSF-HK (Orange Star/Cross): The functional-connectivity representations transformed by GSF-FO and GSF-HK showed broadly similar performance trajectories across the ERR range. At full electrode density, their classification accuracies were generally comparable to those of the DIF-transformed and original untransformed representations, whereas performance gradually decreased as ERR was reduced. Under the adaptive electrode-retention setting, both GSF-based representations remained competitive around ERR = 0.5 and 0.3. Under the PLV condition, however, the GSF-HK-transformed representation showed lower classification accuracy than the baseline under some reduced-electrode settings. Overall, the classification advantage observed for the DIF-transformed representations across the moderate ERR range was less consistently observed for the two GSF-based representations. Under severe electrode reduction, their classification accuracies decreased to approximately 50–56% at ERR = 0.1.

Overall: The results indicate a distinction between the functional-connectivity-level and signal-level transformation strategies. The functional-connectivity representations transformed by DIF and GSF generally maintained relatively high classification performance when the full network or moderately reduced networks were used, whereas the representations obtained after CSD and SSD tended to show lower classification accuracy at these higher electrode densities. This pattern may indicate that signal-level transformation modifies components of the EEG signals that remain relevant to downstream classification, whereas functional-connectivity-level transformation operates directly on the constructed network representation and may preserve more of the discriminative structure present in the originally observed functional networks.

Conversely, the functional-connectivity representations obtained after the signal-level methods, particularly SSD, showed greater stability under substantial electrode reduction. Because CSD and SSD are applied to the EEG signals before functional-connectivity estimation, their effects are incorporated into the subsequently constructed connectivity representations before reduced-electrode networks are evaluated. This processing order may contribute to the relatively stable classification performance observed when fewer electrodes are retained. In contrast, post-hoc functional-connectivity-level transformations such as DIF and GSF operate on the structure of the constructed functional networks and may therefore become more sensitive to reductions in the available network information as electrode density decreases. Overall, the results suggest complementary performance characteristics across electrode-retention conditions: the DIF-transformed representations generally showed favorable classification performance at full to moderate electrode densities, whereas the signal-level approaches, particularly SSD, showed greater stability under highly sparse electrode configurations.

#### 7.1.2. Case Study and Stability Analysis at a Representative Sparsity (ERR = 0.3; SEED)

**Figure 5:**
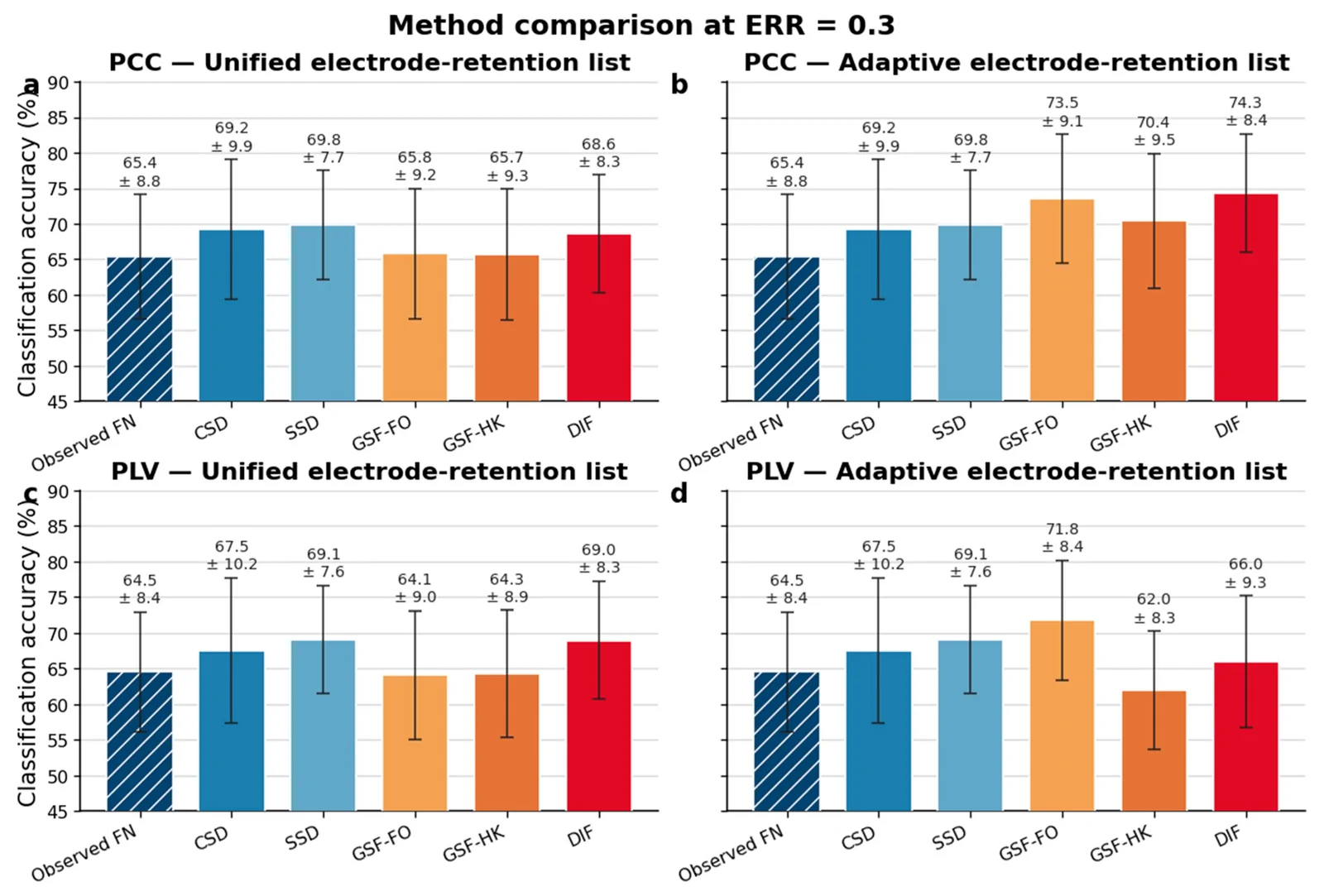
Average classification accuracy (*n* = 30, error bars: 95% CI) achieved by the different methods under ERR = 0.3, across functional connectivity metrics, and electrode-retention strategies. (**a**) PCC with a unified electrode-retention list. (**b**) PCC with an adaptive electrode-retention list. (**c**) PLV with a unified electrode-retention list. (**d**) PLV with an adaptive electrode -retention list.

To further examine the localized behavior of the evaluated functional-connectivity representations under a practically relevant sparse-electrode configuration, a case study was conducted at ERR = 0.3 (Figure 5).

Across all four configurations, the DIF-transformed representations achieved higher mean classification accuracy than the originally observed functional networks. For PCC, classification accuracy increased from 65.4% ± 8.8% to 68.6% ± 8.3% under the unified electrode-retention strategy (Figure 5a) and from 65.4% ± 8.8% to 74.3% ± 8.4% under the adaptive strategy (Figure 5b). For PLV, classification accuracy increased from 64.5% ± 8.4% to 69.0% ± 8.3% under the unified strategy (Figure 5c), whereas a smaller increase, from 64.5% ± 8.4% to 66.0% ± 9.3%, was observed under the adaptive strategy (Figure 5d). These results show that the DIF-transformed representations maintained higher mean classification accuracy than the untransformed baseline when 30% of the electrodes were retained, although the magnitude of the difference varied with the connectivity metric and electrode-retention strategy.

For PCC, the difference between the unified and adaptive electrode-retention strategies was particularly apparent for the DIF-transformed representations. Under the unified strategy, DIF achieved a mean accuracy of 68.6%, slightly lower than CSD (69.2%) and SSD (69.8%) but higher than GSF-FO (65.8%) and GSF-HK (65.7%). Under the adaptive strategy, the DIF-transformed representation achieved the highest mean accuracy among the evaluated conditions (74.3%), followed closely by GSF-FO (73.5%). This pattern suggests that the classification performance of functional-connectivity-level transformations may depend on the electrode-retention strategy used to construct the reduced networks.

A different pattern was observed for PLV. Under the unified strategy, the DIF-transformed representation achieved a mean accuracy of 69.0%, closely matching SSD (69.1%) and exceeding CSD (67.5%), GSF-FO (64.1%), GSF-HK (64.3%), and the originally observed functional network (64.5%). Under the adaptive strategy, however, GSF-FO achieved the highest mean accuracy (71.8%), followed by SSD (69.1%) and CSD (67.5%), whereas the DIF-transformed representation achieved 66.0%. Thus, in contrast to the PCC condition, the adaptive electrode-retention strategy was not associated with a consistent improvement in classification performance for the DIF-transformed PLV representations.

Variability across recordings also differed among the evaluated representations, but no representation showed consistently lower variability across all four configurations. At ERR = 0.3, the standard deviations associated with the DIF-transformed representations ranged from approximately 8.3% to 9.3%, broadly comparable to those of the other evaluated conditions. Accordingly, this case study does not provide evidence for a general reduction in classification variability after DIF transformation. Its main observed benefit at this sparsity level was the higher mean classification accuracy relative to the original untransformed representation, most notably for PCC under the adaptive electrode-retention condition.

#### 7.1.3. Case Study of Robustness to Random Seeds at a Representative Sparsity (ERR = 0.3; SEED)

**Figure 6:**
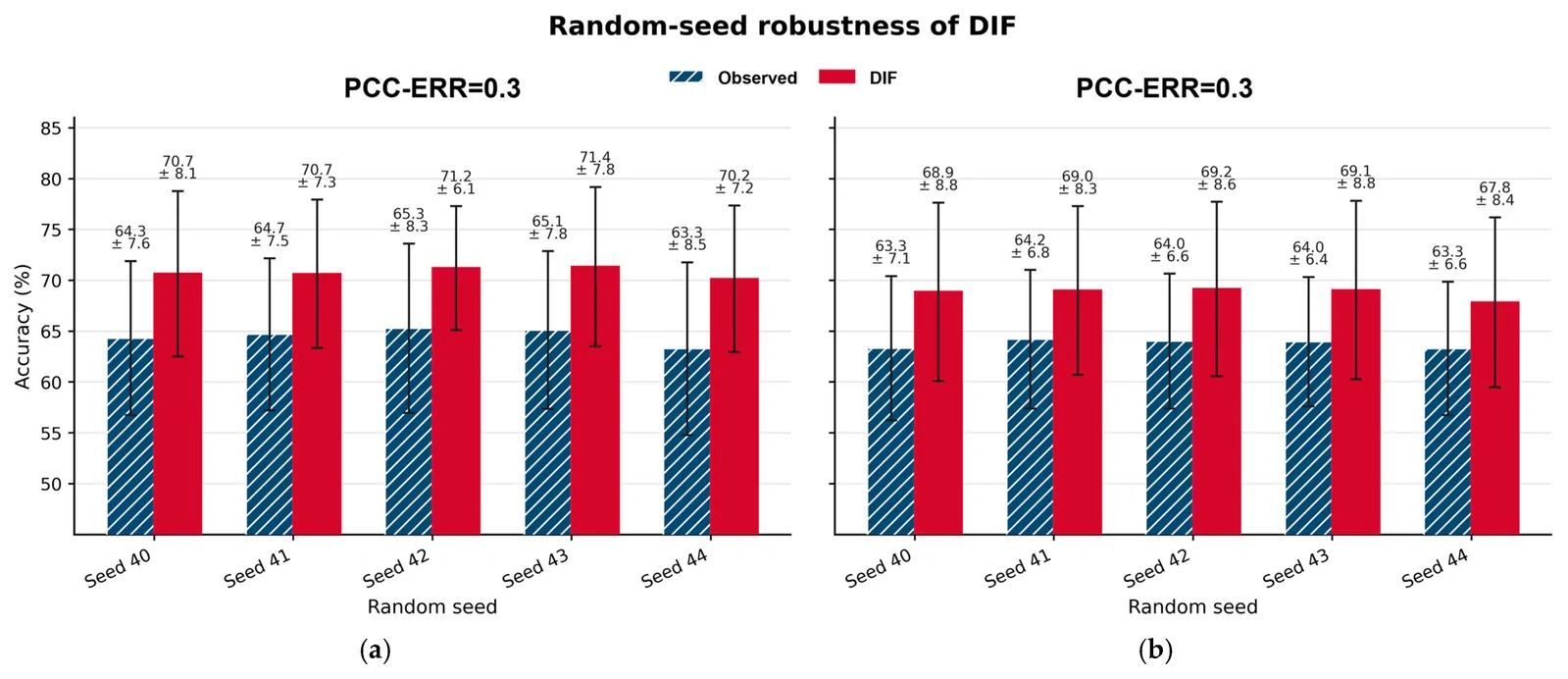
Random-seed robustness of DIF at ERR = 0.3. Classification accuracies of the original and DIF-transformed functional networks across random seeds for (**a**) PCC and (**b**) PLV.

To assess sensitivity to training randomness, the originally observed and DIF-transformed functional-connectivity representations were evaluated using five random seeds on the first session of each of the 10 evaluation subjects. A single session per subject was used to avoid additional within-subject dependence across repeated sessions and to maintain a subject-level paired evaluation framework. Across the five random seeds, the DIF-transformed representations consistently achieved higher classification accuracy than the untransformed baseline, with mean seed-wise differences of 6.26 ± 0.39% for PCC and 5.02 ± 0.38% for PLV (Figure 6a,b).

For statistical inference, classification accuracies were first averaged across the five seeds within each subject, yielding 10 subject-level paired observations for each connectivity measure. Two-sided paired t-tests showed significant positive paired differences for both PCC (Holm-adjusted *p* = 0.040, *dz* = 0.89) and PLV (Holm-adjusted *p* = 0.040, *dz* = 0.77) (Table 3). These results indicate that the higher classification performance associated with the DIF-transformed representations at ERR = 0.3 was consistently observed across the evaluated random seeds.

#### 7.1.4. Area Under the Curve (AUC) Analysis for Accuracy Versus ERRs (SEED)

**Figure 7:**
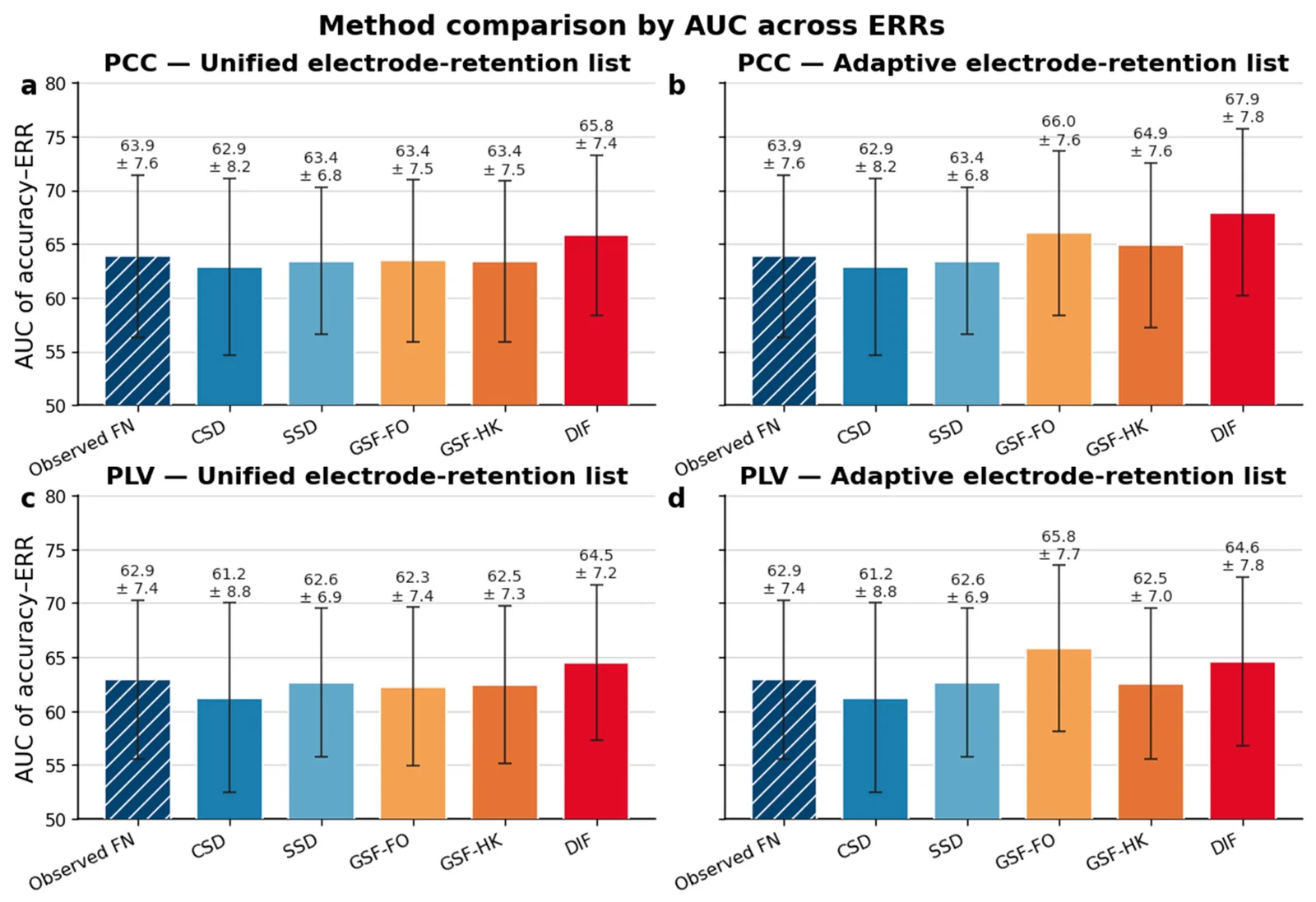
AUC (*n* = 30, error bars: 95% CI) achieved by the different methods, calculated using accuracy versus ERRS (Figure 4), across functional connectivity metrics, and electrode-retention strategies. (**a**) PCC with a unified electrode-retention list. (**b**) PCC with an adaptive electrode-retention list. (**c**) PLV with a unified electrode -retention list. (**d**) PLV with an adaptive electrode -retention list.

To provide a cumulative evaluation of classification performance across the full range of electrode-retention rates, the Area Under the Accuracy-ERR Curve (Accuracy-ERR AUC) was calculated for each evaluated functional-connectivity representation. This metric treats classification accuracy as a function of ERR, as shown in Figure 4, and summarizes performance across the progressive electrode-reduction process. Higher AUC values indicate higher overall classification accuracy maintained across the evaluated ERR range. Figure 7 presents the mean AUC values and corresponding standard deviations across the 30 recording-level classification experiments from the 10 evaluation subjects.

Overall, the DIF-transformed representations generally achieved higher AUC values than the original untransformed functional networks and remained competitive with the other evaluated representations across the ERR range.

For PCC, the DIF-transformed representations achieved the highest mean AUC under both electrode-retention strategies. Under the unified electrode-retention list, DIF achieved a mean AUC of 65.81 ± 7.43, compared with 63.85 ± 7.56 for the originally observed functional networks, and also exceeded the mean AUC values of the competing methods. Under the adaptive strategy, DIF achieved a mean AUC of 67.94 ± 7.78, followed by GSF-FO (66.00 ± 7.64) and GSF-HK (64.90 ± 7.65). These results show that, for PCC, the higher classification performance associated with the DIF-transformed representations was maintained across the evaluated electrode-retention conditions, with the largest cumulative performance observed under adaptive electrode retention.

For PLV, the DIF-transformed representations also showed favorable cumulative classification performance. Under the unified electrode-retention list, DIF achieved the highest mean AUC of 64.51 ± 7.19, compared with 62.91 ± 7.37 for the originally observed functional networks, and exceeded the mean values of the competing methods. Under the adaptive strategy, GSF-FO achieved the highest mean AUC (65.79 ± 7.70), while DIF achieved 64.60 ± 7.80, which remained higher than the original untransformed representation (62.91 ± 7.37). Thus, although the DIF-transformed PLV representations did not yield the highest AUC under every electrode-retention strategy, they consistently showed higher cumulative classification performance than the untransformed baseline across the evaluated conditions.

#### 7.1.5. Subject-Level Statistical Inference for Accuracy–ERR AUC (SEED)

**Figure 8:**
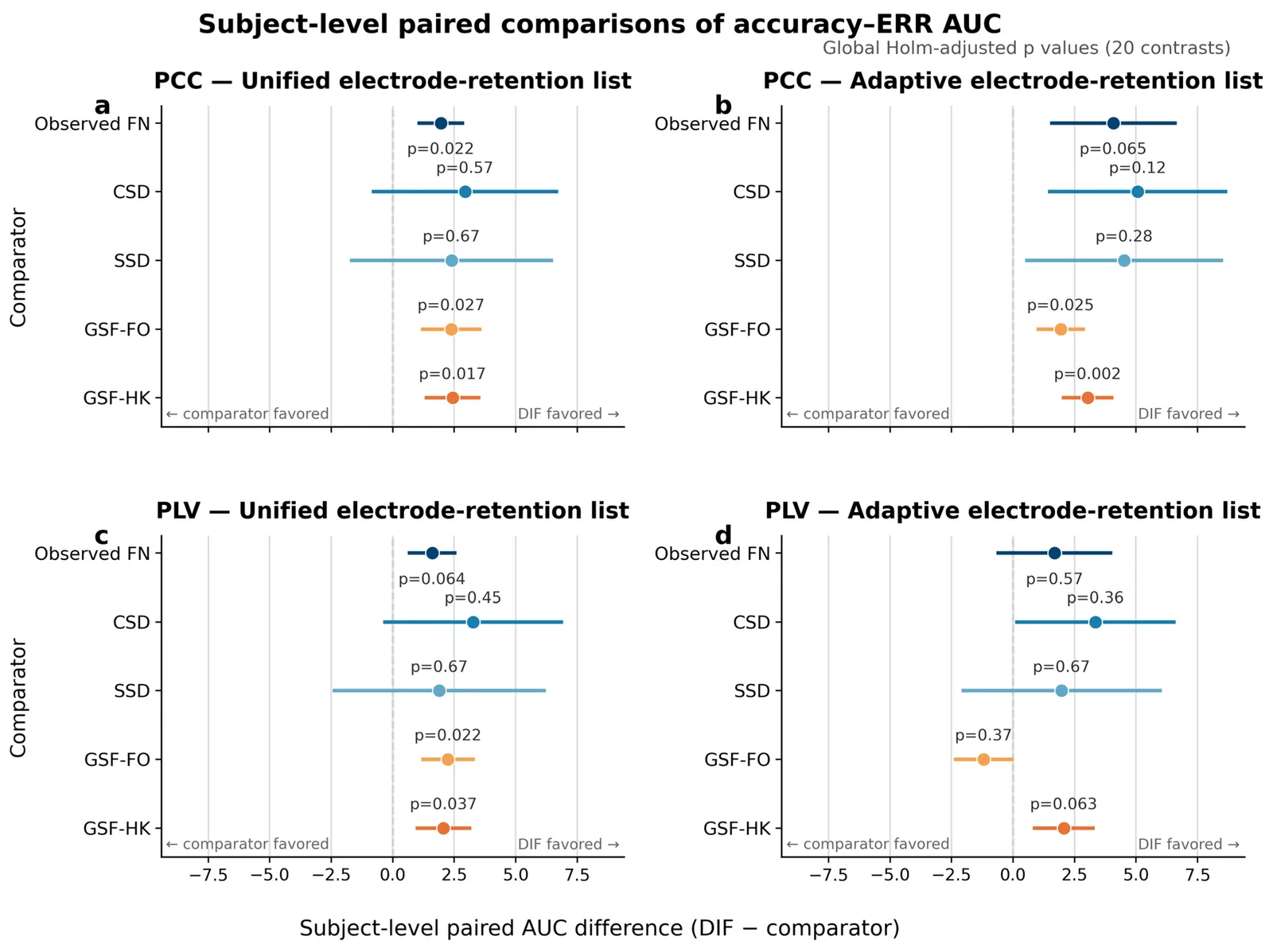
Subject-level paired comparisons of the area under the accuracy–electrode-retention-rate curve (AUC). For each subject, AUCs were calculated separately for the three recordings and then averaged, yielding one AUC per method and subject (*n* = 10). Points represent mean paired AUC differences between DIF and each comparator, and horizontal lines indicate 95% confidence intervals. Positive values favor DIF, whereas negative values favor the comparator; the dashed vertical line denotes no difference. Two-sided paired *t*-tests were performed at the subject level. *p* values were adjusted using the Holm procedure across all 20 prespecified DIF–comparator contrasts from the two connectivity measures and two electrode-retention-list strategies. (**a**–**d**) refer to the combination of PCC, PLV estimation and unified adaptive retention strategy.

To further evaluate the cumulative classification performance associated with the DIF-transformed representations across the full electrode-retention range, subject-level paired comparisons were conducted using the area under the accuracy–ERR curve (AUC). For each subject, AUC values were first calculated separately for the three recordings and then averaged, yielding one subject-level AUC for each evaluated representation (*n* = 10). Two-sided paired t-tests were performed between the DIF-transformed representation and each comparator, followed by a global Holm correction across the 20 prespecified contrasts covering the two connectivity measures and two electrode-retention-list strategies (Figure 8).

For PCC, the DIF-transformed representations showed significant positive AUC differences relative to the observed, GSF-FO, and GSF-HK representations under the unified electrode-retention list (Holm-adjusted *p* = 0.022, 0.027, and 0.017, respectively). Under the adaptive strategy, significant positive differences were observed relative to GSF-FO (*p* = 0.025) and GSF-HK (*p* = 0.002), whereas the difference relative to the observed representation remained positive but did not reach statistical significance after correction (*p* = 0.065).

For PLV, the DIF-transformed representations showed significant positive AUC differences relative to GSF-FO and GSF-HK under the unified strategy (*p* = 0.022 and 0.037, respectively). Under the adaptive strategy, none of the paired comparisons remained statistically significant after Holm correction.

Overall, the subject-level analysis indicates that the higher cumulative classification performance associated with the DIF-transformed representations was most consistently observed relative to the original and GSF-transformed representations, particularly for PCC. In contrast, the paired differences relative to the CSD- and SSD-based representations did not reach statistical significance after correction for multiple comparisons.

#### 7.1.6. Supplementary External Validation Under the Sample-Shuffle Setting (SEED)

In the main evaluation, temporally ordered samples were retained to provide a conservative assessment and reduce the influence of temporal dependence. Although each functional network was independently constructed from a 1-s EEG segment and treated as an individual classifier input, consecutively generated networks may remain temporally correlated because they originate from the same continuous recording and emotional episode. Randomly shuffling these samples before fold partitioning may therefore distribute temporally related networks across the training and test sets, potentially producing a less conservative estimate of generalization.

Nevertheless, sample shuffling is common in EEG classification, while many studies do not report the relevant partitioning details sufficiently clearly to determine whether shuffling was performed. Moreover, some studies treat functional networks estimated from separate, particularly non-overlapping, time windows as individual samples, on the grounds that short-term functional connectivity represents transient and potentially distinct network configurations; under this view, these networks may be regarded as approximately independent for classification purposes, although temporal dependence cannot be completely excluded.

We therefore included this setting as a supplementary validation to improve comparability with the existing literature and examine whether the relative benefit of DIF depends on sample ordering.

**Figure 9:**
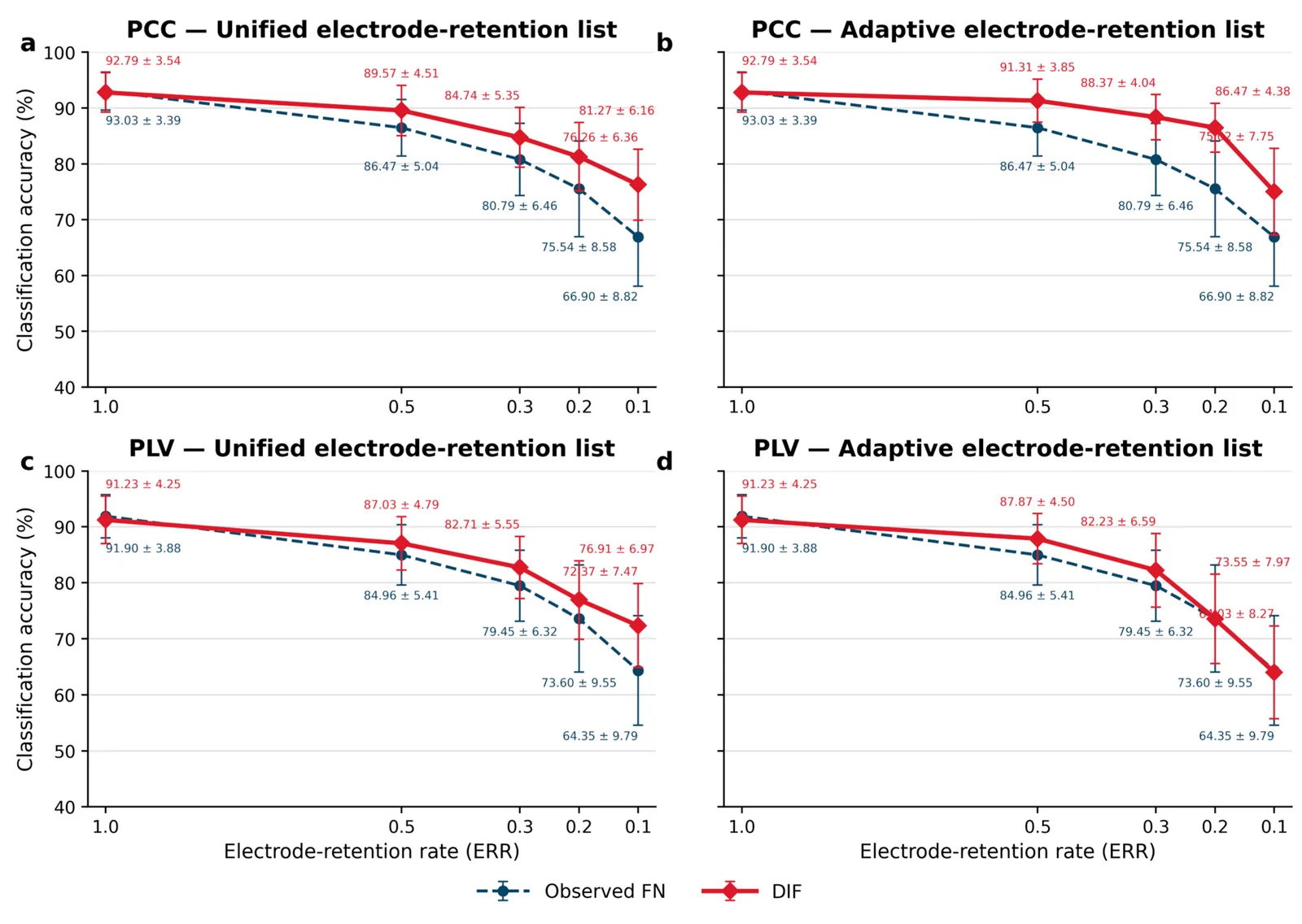
Average classification accuracy across 30 recordings (error bars indicate 95% CIs) for the observed and DIF-transformed functional networks at different electrode-retention rates (ERRs) under the sample-shuffling setting: (**a**) PCC with a unified electrode-retention list; (**b**) PCC with adaptive electrode-retention lists; (**c**) PLV with a unified electrode-retention list; and (**d**) PLV with adaptive electrode-retention lists.

As shown in Figure 9, the DIF-transformed representations generally achieved higher classification accuracy than the original untransformed functional networks across the reduced-electrode conditions. The difference was particularly apparent for PCC, reaching 10.93 percentage points at ERR = 0.2 under the adaptive electrode-retention strategy. For PLV, the largest observed difference under the unified strategy was 8.02 percentage points at ERR = 0.1. Although the absolute classification results obtained under sample shuffling should be interpreted with appropriate caution, the higher classification accuracy associated with the DIF-transformed representations was also observed under this alternative sample-partitioning setting.

### 7.2. Supplementary External Validation on DREAMER Dataset

To further examine the generalizability of DIF on an independent dataset, supplementary experiments were conducted on the DREAMER dataset for valence classification. Table 4 summarizes the subject-level results for PCC and PLV under unified and adaptive electrode-retention lists.

Overall, the DIF-transformed representations showed only small differences in classification accuracy relative to the original untransformed functional networks. For PCC, positive mean differences were observed across all evaluated conditions, ranging from 0.11 to 1.22 percentage points. For PLV, the largest positive difference was observed under the adaptive electrode-retention strategy at ERR = 0.5 (+2.24 percentage points), whereas little difference was observed at ERR = 0.3. However, none of the subject-level paired comparisons reached statistical significance (all *p* > 0.05).

These supplementary results indicate that the DIF-transformed representations achieved classification performance broadly comparable to that of the original untransformed functional networks on the independent DREAMER dataset, with small positive mean differences observed under several conditions but without statistically significant evidence of improvement.

## 8. Additional Analysis of Structural Changes Induced by DIF

**Figure 10:**
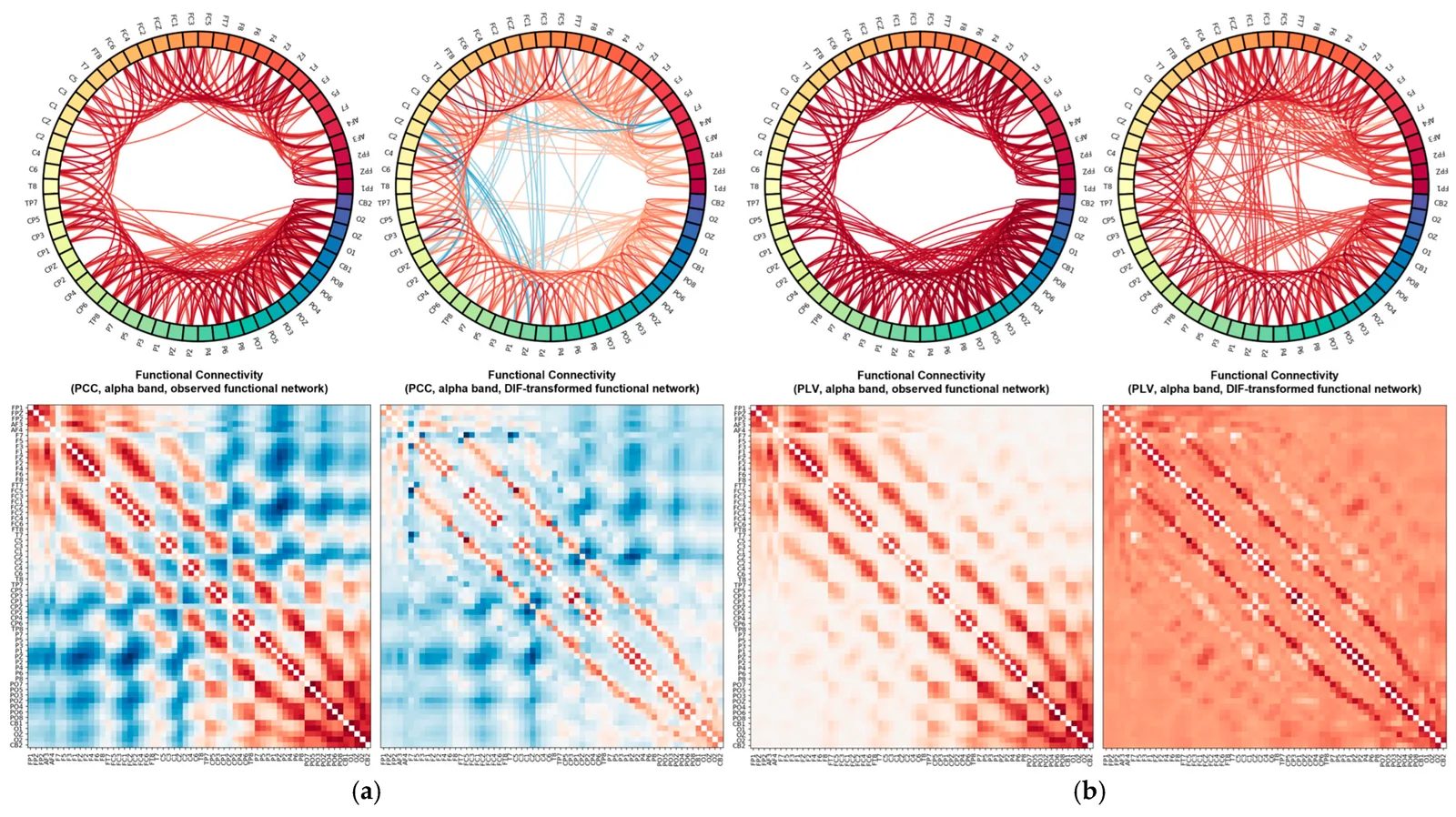
Average functional-connectivity representations and corresponding circle maps (top 300 connections) for the observed and DIF-transformed representations based on alpha-band functional connectivity. Connectivity representations were averaged across all recordings. (**a**) PCC-derived representations. (**b**) PLV-derived representations. The color scale ranges from blue at −1, through white at 0, to red at 1. The diagonal entries were set to NaN to highlight connectivity patterns. FP: frontopolar; AF: anterior frontal; F: frontal; Fz: frontal midline; FT: fronto-temporal; T: temporal; C: central; TP: temporo-parietal; CP: centro-parietal; P: parietal; PO: parieto-occipital; O: occipital; CB: cerebellar.

To illustrate the structural changes introduced by DIF, alpha-band functional-connectivity matrices were selected as an example. The observed functional-connectivity matrices were averaged across the dataset, after which DIF was applied to the resulting average matrices. Figure 10 presents the observed and DIF-transformed matrices, together with connectivity circle maps showing the 300 connections with the largest absolute values.

The alpha band was selected because lower-frequency EEG activity generally exhibits relatively smooth spatial profiles [19,68]. It therefore provides a suitable example for visualizing how a spatially informed transformation modifies locally dominant and spatially distributed matrix patterns. However, this selection does not imply that the observed local patterns can be attributed exclusively to volume conduction.

As shown in Figure 10a,b, the observed representations are characterized by dense short-range patterns and strong values near the matrix diagonal. Following DIF transformation, these local patterns become less dominant, whereas off-diagonal and longer-range patterns become more visually apparent. These observations describe the structural redistribution produced by DIF and are consistent with its spatial inverse-filtering formulation.

However, the results should not be regarded as evidence that volume-conduction contamination has been removed or that the transformed connections more accurately represent the underlying neural interactions. Furthermore, although the input matrices were estimated using PCC and PLV, the DIF-transformed matrices are treated here as PCC-derived and PLV-derived feature representations rather than conventional PCC or PLV estimates. The present analysis does not establish that the transformed PCC-derived matrices retain all correlation-matrix properties or that the transformed PLV-derived matrices remain bounded and physiologically interpretable as conventional PLV estimates. Accordingly, Figure 10 is intended only to illustrate changes in feature organization produced by DIF.

### 8.1. Differentiation Between PCC-Derived and PLV-Derived Representations

Before DIF transformation, the PCC- and PLV-based matrices exhibit broadly similar dominant spatial patterns, particularly strong short-range and near-diagonal structures. Although PCC and PLV quantify different signal relationships, these metric-specific differences are less visually prominent in the observed representations because both are dominated by local matrix patterns.

After DIF transformation, the PCC-derived and PLV-derived representations become more visually differentiated. The transformed PCC-derived representation is sparser, and its inter-regional structures become more apparent. Negative values are also more visible because they are less obscured by dominant positive local patterns. The transformed PLV-derived representation similarly exhibits reduced local dominance but contains a richer off-diagonal organization, including a larger number of visually prominent longer-range and cross-regional patterns.

These differences indicate that DIF redistributes matrix values differently depending on the input connectivity measure.

### 8.2. Regional Organization of the DIF-Transformed Representations

The matrix visualizations in Figure 10 provide a complementary view of the structural changes shown by the circle maps.

For the PCC-derived representation, values within several diagonal blocks, particularly those corresponding to the frontal and occipital regions, are attenuated following DIF transformation. Although within-region and neighboring-region structures remain prominent, several off-diagonal blocks become more visible, including patterns involving the FC–FP, C–FP, C–F, O–CP, and O–P regions. This increased visibility reflects the reduced dominance of local matrix values rather than a demonstrated increase in physiological long-range connectivity.

A broadly similar redistribution is observed in the PLV-derived representation. Local and neighboring-region patterns become less dominant, while off-diagonal structures become more visually apparent. Compared with the PCC-derived representation, the transformed PLV-derived representation shows more pronounced off-diagonal organization, including band-like patterns involving the P–FC regions. Thus, DIF appears to reveal different structural characteristics in PCC- and PLV-derived feature spaces.

Overall, the visual analysis indicates that DIF reduces the relative prominence of local matrix patterns and increases the visibility of cross-regional structures. These descriptive changes complement the classification results by illustrating how DIF reorganizes the input features.

### 8.3. Contextual Interpretation and Limitations

Within-region and neighboring-region structures remain prominent in both the observed and DIF-transformed representations. These short-range patterns cannot be interpreted solely as volume-conduction artifacts because genuine local neural interactions may also contribute to them. Previous studies have associated interactions involving frontal, frontocentral, central, parietal, and occipital regions with executive control, emotion regulation, sensorimotor processing, attention, and affective visual processing. The present visualization cannot separate these physiological contributions from volume conduction or other sources of spatial covariance.

Several off-diagonal patterns become more visible after DIF transformation, including structures involving the P–FC, FP–FC, O–P, O–CP, C–F, and FP–C regions. Their spatial locations overlap with regions previously implicated in emotion-related cognitive processes. This correspondence provides descriptive context for the transformed feature patterns but does not demonstrate that they represent genuine functional pathways or physiologically valid connectivity estimates.

Therefore, the transformed representations should be interpreted primarily as spatially informed feature representations that exhibit altered local and cross-regional organization and improved utility in downstream classification. The present visualization does not establish that DIF corrects volume conduction, recovers the underlying functional network, or preserves the full mathematical and physiological meaning of the original PCC and PLV measures. Direct assessment of these hypotheses would require additional validation, such as forward-model simulations with known source interactions, comparisons with source-space or leakage-resistant connectivity estimates, and explicit evaluation of the mathematical properties of the transformed matrices.

## 9. Discussion

### 9.1. Major Findings: Classification Performance of DIF-Transformed Representations

Conventional countermeasures for volume conduction and spatial smoothing, including source localization [24,25,26,69], SSD and CSD [33,36], operate on EEG signals or reconstructed sources before functional-connectivity estimation. Source-localization methods generally require an inverse model and may be computationally demanding or sensitive to modeling assumptions, whereas CSD enhances the spatial specificity of scalp potentials through a surface-Laplacian transformation. Once pairwise functional connectivity has been constructed, these signal-level processing cannot be applied without reprocessing the original EEG signals. In contrast, GSP provides a framework for directly filtering out spatially smoothed components in graph-structured functional-connectivity representations [37,38,54,70]. This distinction motivates the investigation of spatially informed transformations that operate directly at the functional-connectivity level, including approaches that do not rely on a GSP framework.

DIF was developed as such a functional-connectivity-level transformation, using a direct spatial-kernel formulation applied to matrix-based functional networks rather than a GSP framework. It models spatial smoothing in functional-connectivity matrices through a diffusion-like approximation based on inter-electrode geometry and applies a regularized inverse operation to redistribute the matrix values. The diffusion model is motivated by spatial-spreading phenomena that may include volume conduction; however, the present experiments do not directly establish that the modeled component corresponds specifically to volume conduction. DIF should therefore be interpreted as a spatially informed functional-connectivity transformation rather than a validated method for volume-conduction correction.

The classification experiments on the SEED dataset showed that DIF-transformed functional networks (a classical functional-connectivity representation) generally achieved higher classification performance than the corresponding originally observed networks. The clearest evidence was obtained for PCC-derived features, for which positive Accuracy–ERR AUC differences were observed under both electrode-retention strategies. At the subject level, this improvement reached statistical significance under the unified strategy for electrode sparsification, while the positive difference under the adaptive strategy did not remain significant after global Holm correction. At ERR = 0.3, the random-seed analysis further showed a significant improvement for PCC after subject-level averaging and multiplicity correction. Taken together, these findings indicate that DIF provides a consistent performance benefit for PCC-derived representations across the evaluated electrode-sparsification conditions, although the strength of statistical evidence varies with the electrode-retention strategy.

For PLV-derived features, the results were more dependent on the electrode- sparsification strategy and comparator. DIF generally improved classification performance relative to the observed PLV baseline, including in the random-seed case study at ERR = 0.3. Nevertheless, the subject-level AUC advantage over the observed PLV baseline did not remain significant after global correction under the unified strategy, and none of the paired comparisons remained statistically significant after Holm correction under the adaptive strategy. Accordingly, DIF should be considered competitive for PLV functional networks, but not consistently superior to the strongest graph-filtering alternatives.

DIF also exhibited relatively small descriptive standard deviations in several experimental settings. This pattern suggests potentially stable classification performance across recordings, but differences in variability were not directly tested. The results therefore support lower observed dispersion in particular conditions rather than a general claim of statistically superior stability.

On the DREAMER dataset, the DIF-transformed networks showed classification performance broadly comparable to that of the corresponding originally observed networks, with small positive mean differences observed under several conditions. However, none of the subject-level paired comparisons reached statistical significance, indicating that the generalizability of DIF across datasets and signal qualities requires further investigation, development, and optimization.

Taken together, the findings demonstrate that DIF can improve the classification utility of functional-connectivity-derived representations, particularly those constructed from PCC, while maintaining competitive performance under electrode sparsification.

### 9.2. Methodological and Engineering Contributions

#### 9.2.1. Spatially Informed Transformation at the Functional-Connectivity Level

The principal methodological contribution of this study is the formulation of a spatially informed transformation that operates directly on functional-connectivity representations. Unlike source localization SSD, and CSD, which modify EEG signals or reconstructed sources before connectivity estimation, DIF can be applied after functional feature construction and requires only the connectivity matrices and electrode coordinates. This positioning makes it compatible with analysis pipelines in which the original EEG signals, individualized head models, or source reconstructions are unavailable.

The study also formulates GSF-FO and GSF-HK as bilateral transformations of functional networks, thereby providing functional-connectivity-level comparators within a common spatial modeling framework. DIF, GSF-FO, and GSF-HK differ in their filtering responses, but all use inter-electrode geometry to transform the row and column dimensions of the connectivity representations. Their performance relative to the untransformed baseline indicates that incorporating spatial structure at the functional-connectivity level can improve downstream feature representations under some conditions.

These results demonstrate the potential of functional-connectivity-level spatial modeling as a representation-transformation strategy for classification. Within this interpretation, DIF provides a regularized inverse-diffusion formulation that is particularly effective for PCC-derived representations, whereas its relative performance for PLV-derived representations depends on the sparsification strategy and the competing graph filter.

#### 9.2.2. Practical Implications for BCI Systems

The characteristics of DIF may provide several practical advantages for connectivity-based BCI applications. Unlike source-reconstruction-based approaches, DIF does not require MRI data or individualized head models and can be applied directly using electrode-coordinate information. This relatively simple requirement may facilitate its use in lightweight systems, cost-constrained applications, and legacy EEG datasets in which subject-specific anatomical information is unavailable.

Compared with the original untransformed functional networks, the DIF-transformed representations generally achieved higher classification accuracy for both PCC- and PLV-derived features across the evaluated conditions, although the magnitude and statistical significance of the differences varied with connectivity measure, electrode-retention strategy, and dataset. Compared with conventional signal-level approaches such as CSD and SSD, DIF provides an alternative that operates directly on already constructed functional-connectivity representations. This property may be particularly useful in connectivity-based BCI pipelines where only functional-network features are available or where signal-level reprocessing is impractical.

### 9.3. Limitations and Future Directions

Several limitations should be noted. DIF depends on electrode spatial geometry, and its effectiveness may vary with electrode-coordinate accuracy and nonstandard montage configurations. Moreover, the Gaussian diffusion kernel adopted here may not fully represent the spatial patterns present in functional-connectivity matrices, and dynamic or frequency-specific diffusion processes were not examined. Future work may therefore improve the diffusion kernel, refine the distance matrix or its spatial representation, and develop more expressive models of the diffusion and inverse processes, together with more systematic parameter optimization.

Beyond methodological refinement, the present findings also suggest broader future directions, including the extension of additional signal-level methods to the functional-connectivity level, a more comprehensive investigation of performance–efficiency trade-offs for BCI applications, and feature fusion between amplitude-based and phase-based connectivity patterns, given the structural differences observed between the DIF-transformed functional network derived from PCC and PLV.

The evaluation framework employed in this study assesses whether DIF-transformed functional networks exhibit improved discriminative power in classification experiments. This evaluation strategy is aligned with the objectives of engineering and BCI applications, where decoding performance is an important measure of practical utility. However, it does not directly verify whether DIF mitigates volume conduction or whether the transformed representations more accurately reflect the underlying neural interactions. Such validation would require independent evidence, for example through comparisons with synthetic or forward-model simulations, leakage-resistant connectivity measures, MEG-based connectivity estimates, or source-space connectivity reconstructed using inverse modeling. Therefore, the findings should be interpreted primarily as evidence that DIF improves the practical utility of functional-connectivity-derived representations for classification. Further research is needed to evaluate the mathematical properties and physiological significance of the transformed representations and to clarify their relationship to volume conduction and the underlying neural mechanisms.

## Figures and Tables

**Table 1:** Deep-learning architecture.

Stage	Operation	Details
Input	Input + Per-channel Instance Normalization	Collection of functional networks *N* × *B* × *C* × *C* Note: Normalization is performed independently within each 62 × 62 feature map.
Conv Layer 1	Conv + BN + ReLU	3 × 3, 32 filters, stride = 1, padding = 1
Pooling 1	Max Pooling	3 × 3, stride = 3
Conv Layer 2	Conv + BN + ReLU	3 × 3, 64 filters, stride = 1, padding = 1
Pooling 2	Adaptive Max Pooling	Output size = 1 × 1 (Global pooling)
Flatten	Flatten	Convert to 1D vector (64)
FC Layer 1	Fully Connected	32 neurons
FC Layer 2	Fully Connected	3 neurons
Output	SoftMax	3-category classification (positive-neutral-negative according to SEED dataset)

**Table 2:** Parameter Settings for Different Methods.

Method	Optimal Parameters
SEED Dataset	
Proposed DIF	σ: 0.161 (k-NN, *k* = 3); λ: 0.0189 (L-curve)
Competitor 1: GSLF	σ: 0.161 (k-NN, *k* = 3)
Competitor 2: GSF-FO	σ: 0.161 (k-NN, *k* = 3); High pass
Competitor 3: GSF-HK	σ: 0.161 (k-NN, *k* = 3); High pass
DREAMER Dataset	
Proposed DIF	σ: 0.297 (k-NN, *k* = 3); λ: 0.0418 (L-curve)
Competitor 1: GSLF	σ: 0.297 (k-NN, *k* = 3)
Competitor 2: GSF-FO	σ: 0.297 (k-NN, *k* = 3); High pass
Competitor 3: GSF-HK	σ: 0.297 (k-NN, *k* = 3); High pass

**Table 3:** Subject-level paired statistical analysis of random-seed robustness at ERR = 0.3.

Measure	Acc. (Observed)	Acc. (DIF)	Difference	95% CI	*p* Holm	*dz*
PCC	64.55 ± 8.27%	70.81 ± 7.60%	+6.26 pp	[1.24, 11.28]	0.0399	0.89
PLV	63.78 ± 6.94%	68.80 ± 8.95%	+5.02 pp	[0.37, 9.66]	0.0399	0.77

**Table 4:** Subject-level external validation results on the DREAMER dataset.

ERL	Measure	ERR	Acc. (Observed)	Acc. (DIF)	Difference	95% CI	dz	*p*
Unified	PCC	1	64.38 ± 13.27	65.51 ± 12.46	+1.13	[−0.43, 2.69]	0.40	0.143
Unified	PCC	0.5	64.19 ± 13.26	64.30 ± 13.40	+0.11	[−1.42, 1.64]	0.04	0.882
Unified	PCC	0.3	63.36 ± 13.99	63.64 ± 13.94	+0.28	[−0.91, 1.47]	0.13	0.621
Unified	PLV	1	64.43 ± 12.52	64.90 ± 12.41	+0.47	[−0.76, 1.70]	0.21	0.424
Unified	PLV	0.5	62.83 ± 13.47	63.85 ± 13.49	+1.02	[−0.39, 2.43]	0.40	0.143
Unified	PLV	0.3	63.00 ± 12.97	63.00 ± 12.97	−0.00	[−1.26, 1.26]	−0.00	1.000
Adaptive	PCC	0.5	64.19 ± 13.26	65.18 ± 12.01	+0.99	[−1.86, 3.84]	0.19	0.467
Adaptive	PCC	0.3	63.36 ± 13.99	64.58 ± 12.26	+1.22	[−2.03, 4.48]	0.21	0.434
Adaptive	PLV	0.5	62.83 ± 13.47	65.06 ± 12.30	**+2.24**	[−1.32, 5.79]	0.35	0.198
Adaptive	PLV	0.3	63.00 ± 12.97	62.92 ± 11.25	−0.08	[−4.01, 3.85]	−0.01	0.965

## Data Availability

The original contributions related to parameter optimization and classification performance evaluation presented in this study are included in the article. Further inquiries can be directed to the corresponding author(s). Restrictions apply to the availability of the EEG dataset used in this study. The data were obtained from the SEED team and are available from the SEED project [71] with permission from the dataset developers.
